# Pathogenomics of Virulence Traits of *Plesiomonas shigelloides* That Were Deemed Inconclusive by Traditional Experimental Approaches

**DOI:** 10.3389/fmicb.2018.03077

**Published:** 2018-12-21

**Authors:** Temitope C. Ekundayo, Anthony I. Okoh

**Affiliations:** ^1^SAMRC Microbial Water Quality Monitoring Centre, University of Fort Hare, Alice, South Africa; ^2^Applied and Environmental Microbiology Research Group, Department of Biochemistry and Microbiology, University of Fort Hare, Alice, South Africa; ^3^Department of Biological Sciences, University of Medical Sciences, Ondo City, Nigeria

**Keywords:** *Plesiomonas shigelloides*, virulence factor, antibiotic resistance, bioinformatics, pathogenicity island

## Abstract

One of the major challenges of modern medicine includes the failure of conventional protocols to characterize the pathogenicity of emerging pathogens. This is particularly apparent in the case of *Plesiomonas shigelloides*. Although a number of infections have been linked to this microorganism, experimental evidence of its virulence factors (VFs), obtained by traditional approaches, is somewhat inconclusive. Hence, it remains unclear whether *P. shigelloides* is a true or opportunistic one. In the current study, four publicly available whole-genome sequences of *P. shigelloides* (GN7, NCTC10360, 302-73, and LS1) were profiled using bioinformatics platforms to determine the putative candidate VFs to characterize the bacterial pathogenicity. Overall, 134 unique open reading frames (ORFs) were identified that were homologous or orthologous to virulence genes identified in other pathogens. Of these, 52.24% (70/134) were jointly shared by the strains. The numbers of strain-specific virulence traits were 4 in LS1; 7 in NCTC10360; 10 in 302-73; and 15 in GN7. The pathogenicity islands (PAIs) common to all the strains accounted for 24.07% ORFs. The numbers of PAIs exclusive to each strain were 8 in 302-73; 11 in NCTC10360; 14 in GN7; and 18 in LS1. A PAI encoding *Vibrio cholerae* ToxR-activated gene d protein was specific to 302-73, GN7, and NCTC10360 strains. Out of 33 antibiotic multi-resistance genes identified, 16 (48.48%) genes were intrinsic to all strains. Further, 17 (22.08%) of 77 antibiotic resistance islands were found in all the strains. Out of 23 identified distinct insertion sequences, 13 were only harbored by strain LS1. The number of intact prophages identified in the strains was 1 in GN7; 2 in 302-73; and 2 in NCTC10360. Further, 1 CRISPR element was identified in LS1; 2 in NCTC10360; and 8 in 302-73. Fifteen (78.95%) of 19 secretion systems and secretion effector variants were identified in all the strains. In conclusion, certain *P. shigelloides* strains might possess VFs associated with gastroenteritis and extraintestinal infections. However, the role of host factors in the onset of infections should not be undermined.

## Introduction

*Plesiomonas shigelloides* (hereafter *Plesiomonas*) possesses various VFs that result in its emerging pathogen status. E.g., the pathophysiology of *Plesiomonas* in certain infections has been attributed to possible endotoxin production by this bacterium (Okawa et al., 2004; Kaszowska et al., 2016). Endotoxins have been proposed to be central to septic shock and sepsis associated with plesiomonad infections (Alexander and Rietschel, 2001), and to biofilm formation, attachment, invasion of eukaryotic host cells, complement-resistance in the serum, or blood (Aquilini et al., 2013). Other reported virulence attributes ascribed to plesiomonads include the production of cytotoxic lipopolisaccharide complex (Fisher et al., 1988; Olsvik et al., 1990; Abbott et al., 1991; Ekman, 2003; Falcón et al., 2003; Okawa et al., 2004), β-hemolysin (Aldová et al., 1966; Zajc-Satler et al., 1972; Janda and Abbott, 1993; Santos et al., 1999; Krovacek et al., 2000; Baratéla et al., 2001; Falcón et al., 2003), enterotoxins (Sanyal et al., 1975, 1980; Saraswathi et al., 1983; Matthews et al., 1988; Sears and Kaper, 1996), adherence factors (Schubert and Holz-Bremer, 1999; Theodoropoulos et al., 2001; Ekman, 2003; Tsugawa et al., 2005), invasion factors (Sanyal et al., 1980; Binns et al., 1984; Olsvik et al., 1985; Herrington et al., 1987; Theodoropoulos et al., 2001; Tsugawa et al., 2005), cytolysin (Gurwith and Williams, 1977; Sanyal et al., 1980; Gardner et al., 1987; Olsvik et al., 1990; Abbott et al., 1991; Dumontet et al., 1998; Ekman, 2003), elastin (Ingram et al., 1987; Nolte et al., 1988; Rust et al., 1994; Santos et al., 1999), plasmids (Holmberg and Farmer, 1984; Herrington et al., 1987; Fisher et al., 1988; Nolte et al., 1988; Olsvik et al., 1990; Abbott et al., 1991; Taylor et al., 1993; Marshall et al., 1996; Bravo et al., 1998; Shepherd et al., 2000; Avison et al., 2001; Kubler-Kielb et al., 2008), tetrodotoxin (Wei et al., 1994; Cheng et al., 1995), and histamine (Lopez-Sabater et al., 1996; Dewaal et al., 2006; Bjornsdottir-Butler et al., 2011).

In addition, antibiotic and metal resistance as single or multiple resistance traits (Meyers et al., 1976; Brenden et al., 1988; Fisher et al., 1988; Avison et al., 2000; Stock and Wiedemann, 2001a) or acquired traits (Kain and Kelly, 1989a; Clark et al., 1990; Olsvik et al., 1990; Yeh and Tsai, 1991; Shaw et al., 1993; Bravo-Fariñas et al., 1998; González et al., 1999; Avison et al., 2000; Wong et al., 2000; Stock and Wiedemann, 2001b; Suthienkul et al., 2001; González-Rey et al., 2004; Jun et al., 2011) of plesiomonads have been reported.

*Plesiomonas* have been credited with causing many infections over the years. These include gastroenteritis and various forms of diarrhea (Tsukamoto et al., 1978; Wong et al., 2000; Chakour et al., 2002; Klontz et al., 2012; Pfeiffer et al., 2012; Novoa-Farías et al., 2016). Of major concern are extraintestinal infections of the central nervous system, such as neonatal meningoencephalitis, meningitis, sepsis, and septic shock (Claesson et al., 1984; Kalotychou et al., 2002; Auxiliadora-Martins et al., 2010; Ozdemir et al., 2010; Xia et al., 2015; Bowman et al., 2016). Other infections include peritonitis (Alcañiz et al., 1995), cellulitis (Gopal and Burns, 1991; Jönsson et al., 1998), wound and foot infections (McCracken and Barkley, 1972; Herve et al., 2007; Pence, 2016), endophthalmitis (Butt et al., 1997; Mahrshmann and Lyons, 1998), and keratitis (Butt et al., 1997; Klatte et al., 2012). Further, pneumonia (Schneider et al., 2009), migratory polyarthritis (Gupta, 1995), cholecystitis (Claesson et al., 1984; Kennedy et al., 1990), pyosalpinx (Roth et al., 2002), pseudo-appendicitis (Fischer et al., 1988), peritonitis (Patel et al., 2016), cholangitis and pancreatitis (Kennedy et al., 1990), and septicemia (Clark et al., 1991) have been attributed to *Plesiomonas*. However, determination of the associated virulence markers and pathogenic strains has been challenging and frustrating. While the majority of traditional approaches yielded inconclusive data with respect to the bacterium’s pathogenicity, others concluded that the virulence potential of the microorganism is low (Olsvik et al., 1990; Abbott et al., 1991).

Furthermore, suitable models for studying the pathogenesis of *Plesiomonas* have not yet been established and traditional models proved to be inappropriate. The development of polymerase chain reaction and other rapid low-cost protocols, already used for the analysis of other enteropathogens, for the detection of *Plesiomonas* VFs is also urgently required. Presently, no specific primer- and molecular-based techniques for the detection of the major, if not all, predicted *Plesiomonas* VFs exist. Further, strain-specific and rapid systems for the delineation of pathogenic and non-pathogenic *Plesiomonas* strains are lacking.

Since the emergence of new pathogens (including *Plesiomonas*) is linked to complex interrelated factors, of which mutation and horizontal gene transfer are the focal driving elements (Che et al., 2014), it is not implausible that several such events occurred in *Plesiomonas*. In most cases, gene acquisition or loss could impact genome evolution (Che et al., 2014), converting a non-pathogenic strain into a pathogenic one (McDaniel and Kaper, 1997). Although these phenomena may have contributed to the emergence of pathogenicity in *Plesiomonas*, no studies focusing on this issue have been reported. The study of PAIs, resistance islands, metabolic islands, mobility genes, IS elements, phage-related genes, tRNA genes, and direct repeats will be important for the characterization of the virulence and genome evolution of *Plesiomonas.*

Consequently, rapid specific culture-independent tools for pathogenicity characterization should be developed for *Plesiomonas*. A comparative *in silico* profiling of the available genomes may provide important clues prior to wet-lab studies for the assessment of virulence marker candidates in *Plesiomonas* and answer other questions about its emerging pathogenicity capabilities. This would not only reveal the as yet unknown virulence traits associated with specific strains, but also inform the development of rapid and inexpensive tools for strain screening and diagnostic characterization of plesiomonads. In the current study, we therefore profiled the publicly available complete genome sequence assemblies of *Plesiomonas in silico* to identify the associated type III, IV, VI, and VII secretion systems (T3SS, T4SS, T6SS, and T7SS, respectively); integrative and conjugation elements (ICE elements); prophages; VFs; ARs; ARIs; type III, IV, VI, and VII secretion effectors (T3SE, T4SE, T6SE, and T7SE, respectively); class I integrons, IS elements; and CRISPR.

## Materials and Methods

### Whole-Genome Sequence Assembly Selection

The four publicly available sequence assemblies of *Plesiomonas* were downloaded from NCBI website via ftp.ncbi.nlm.nih.gov/genomes/ASSEMBLY_BACTERIA (Table 1; last accessed [20 December 2017]).

**Table 1:** Published genome assemblies of *P. shigelloides* (last accessed [20 December 2017]).

Strain	Clade ID	Assembly	Size (Mb)	GC%	WGS	Scaffolds	Genes	Proteins	References
NCTC10360	21595	GCA_900087055.1_31289_E01	3.40598	52	–	1	3038	2871	Alexander et al., 2016
302-73	21595	GCA_000392595.1_PleShi1.0	4.08396	51.1	AQQO01	296	3424	3227	Piqué et al., 2013
LS1	21595	GCA_002093895.1_ASM209389v1	3.86606	51.6	MUNJ01	51	3444	3286	Lei et al., 2017
GN7	21595	GCA_000813415.1_ASM81341v1	3.91623	51.6	JWHQ01	83	3508	3349	Unpublished


### Detection of Virulence Traits

Presence of PVFs within each genome assembly was predicted using the VRprofile 2.0 (Li et al., 2018). For gene clusters (including genes encoding T3SS, T4SS, T6SS, and T7SS), ICE, and prophage prediction, default parameter settings were used. BLASTP searches were done with a minimum *e*-value of 1 × 10^-4^; HMMer search *E*-value of 1 × 10^-4^ for ICE and prophage detection; the minimum number of significant hits for each prophage-like region set to 5; and a minimum length of direct repeats of an ICE-like region of 15 bp. Single genes related to VFs, ARs, T3SE, T4SE, T6SE, T7SE, class I integrons, IS elements, GI-like region, PAIs, and ARIs were predicted by setting the BLAST *Ha*-value to 0.64 (Li et al., 2018). The distribution of PVFs and related genes among the various strains was evaluated using a web-based Venn diagram program^1^.

In addition, a direct search of the WGS for presence of elements related to already suggested virulence mechanisms in *P. shigelloides* such as heme iron uptake systems or iron-siderophores (Daskaleros et al., 1991; Santos et al., 1999; Henderson et al., 2001; Gonzalez-Rodriguez et al., 2007; Oldham et al., 2008; Rodríguez-Rodríguez and Santos, 2018) was performed.

### Detection of Prophage Sequences

To identify prophages within the *Plesiomonas* genomes, strain sequences were analyzed using the PHAge Search Tool Enhanced Release (Zhou et al., 2011; Arndt et al., 2016).

### Determination of the CRISPR Elements

CRISPR elements in the *Plesiomonas* genomes were predicted using CRISPR Finder by Random Forest program (Wang and Liang, 2017).

### Detection of Plasmids

Plasmid elements in the *Plesiomonas* genomes were predicted using PlasmidFinder 1.3 (Carattoli et al., 2014) and a direct search for replicon information on NCBI website^2^.

## Results

**FIGURE 1:**
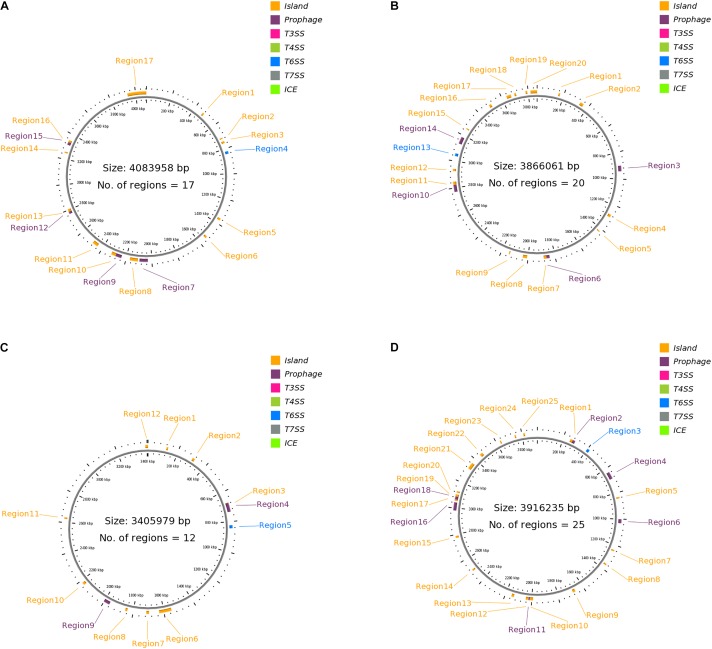
**(A)** Clustered genes related to pathogenicity traits in *P. shigelloides* strain 302-73. Detailed attribute of each region is presented as s/n, location, size (bp), GC% and loci tag/ORF coverage as follows: **1:**470834..483428, 12595, 44.70, ORF2_383-ORF2_394; **2:** 711183..719911, 8729, 40.26, ORF2_576-ORF2_585; **3:** 740699..756945, 16247, 46.49, ORF2_607- ORF2_617; **4:** 822581..842820, 20240, 54.02, ORF2_668-ORF2_682; **5:** 1355512..1371975, 16464, 50.23, ORF2_1092-ORF2_1120; **6:** 1528926..1544581, 15656, 39.02, ORF2_1266-ORF2_1280; **7:** 2030969..2078415, 47447, 49.39, ORF3_214 - ORF3_276; **8:** 2030969..2095574, 64606, 49.46, ORF3_214 - ORF3_291; **9:** 2107619..2170457, 62839, 46.35, ORF5_2 - ORF12_27, **10:** 2237256..2283735, 46480, 50.17, ORF12_83-ORF12_127; **11:** 2284029..2318838, 34810, 44.15, ORF12_128-ORF12_160; **12:** 2440089..2484543, 44455, 44.18, ORF12_277-ORF12_312; **13:** 2768028..2789058, 21031, 50.25, ORF12_549 - ORF12_565; **14:** 2785662..2805932, 20271, 45.82, ORF12_561-ORF12_588; **15:** 3247461..3257433, 9973, 51.77, ORF12_977-ORF12_985; **16:** 3312901..3342556, 29656, 49.84, ORF12_1032-ORF12_1070; **17:** 3333679..3350402, 16724, 46.76, ORF12_1058 - ORF12_1074. **(B)** Clustered genes related to pathogenicity traits in *P. shigelloides* strain LS1. Detailed attribute of each region is presented as s/n, location, size (bp), GC% and loci tag/ORF coverage as follows: **1**: 159754..171539, 11786, 36.75, ORF1_138 - ORF1_152; **2:** 333282..360456, 27175, 38.41, ORF2_2 - ORF2_29, **3:** 900943..942310, 41368, 47.42, ORF3_205 - ORF4_6; **4:** 924180..938968, 14789, 46.44, ORF3_227 - ORF3_250; **5:** 1192553..1222251, 29699, 47.92, ORF4_223 - ORF5_11; **6:** 1273566..1293909, 20344, 41.65, ORF5_50 - ORF5_69; **7:** 1418262..1426747, 8486, 57.02, ORF5_179 - ORF5_188; **8:** 1840332..1883658, 43327, 51.82, ORF8_2 - ORF8_63; **9:** 1863820..1884450, 20631, 50.24, ORF8_30 - ORF8_64; **10:** 2007292..2039280, 31989, 42.29, ORF8_180 - ORF9_6; **11:** 2136454..2144822, 8369, 51.43, ORF9_92 - ORF9_99; **12:** 2770842..2845573, 74732, 52.04, ORF13_39 - ORF13_106; **13:** 2822483..2847354, 24872, 48.26, ORF13_74 - ORF13_108; **14:** 2925054..2941832, 16779, 43.86, ORF14_72 - ORF14_85; **15:** 3036530..3056770, 20241, 54.08, ORF15_54 - ORF15_68; **16**: 3132048..3183899, 51852, 47.08, ORF16_45 - ORF16_103; **17:** 3135832..3161385, 25554, 42.67, ORF16_48 - ORF16_76; **18**: 3251054..3259256, 8203, 45.85, ORF17_58 - ORF17_70; **19**: 3490616..3508713, 18098, 45.13, ORF20_1 - ORF20_18; **20:** 3635149..3669322, 34174, 39.18, ORF22_36 - ORF23_34. **(C)** Clustered genes related to pathogenicity traits in *P. shigelloides* strain NCTC10360. Detailed attribute of each region is presented as s/n, location, size (bp), GC% and loci tag/ORF coverage as follows: **1:** 121244..132239, 10996, 34.23, ORF1_108-ORF1_117; **2:** 303306..319831, 16526, 50.34, ORF1_268-ORF1_296; **3:** 675181..700459, 25279, 46.37, ORF1_586-ORF1_617; **4:** 677319..735911, 58593, 50.75, ORF1_588-ORF1_640; **5:** 823854..844094, 20241, 54.07, ORF1_706 ORF1_720; **6:** 1548988..1630373, 81386, 39.03, ORF1_1310-ORF1_1389; **7:** 1696284..1714355, 18072, 48.88, ORF1_1445-ORF1_1464; **8:**1838362..1852957, 14596, 39.6, ORF1_1583-ORF1_1598; **9:** 1961886..2002266, 40381, 50.19, ORF1_1691-ORF1_1748; **10:** 1983206..2002266, 19061, 45.73, ORF1_1716-ORF1_1748; **11:** 2166969..2184623, 17655, 44.81, ORF1_1884-ORF1_1907; **12:**2623511..2633470, 9960, 51.75, ORF1_2292-ORF1_2300. **(D)** Clustered genes related to pathogenicity traits in *P. shigelloides* strain GN7. Detailed attribute of each region is presented as s/n, location, size (bp), GC% and loci tag/ORF coverage as follows: **1:** 253113...279252, 26140, 46.63, ORF2_172- ORF2_190; **2:** 271577..291757, 20181, 45.45, ORF2_185-ORF2_198; **3:** 398271..418511, 20241, 54.15, ORF4_86 ORF4_100; **4:** 633845..687788, 53944, 51.53, ORF5_172-ORF5_233; **5:** 833184..844394, 11211, 46.01, ORF5_354-ORF5_368; **6:** 1000747..1030898, 30152, 51.06, ORF7_109-ORF7_149; **7:** 1236546..1247816, 11271, 50.27, ORF:ORF9_3-ORF9_26; **8:** 1355229..1366836, 11608, 53.68, ORF:ORF13_2-ORF14_8; **9:** 1664884..1684947, 20064, 43.91, ORF18_40-ORF18_60; **10:** 1990036..2015370, 25335, 40.67, ORF19_207-ORF19_235; **11:** 2015357..2025368, 10012, 52.86, ORF19_234-ORF19_244; **12:** 2027754..2045137, 17384, 39.50, ORF20_2-ORF20_20; **13:** 2135394..2154633, 19240, 39.06, ORF23_1-ORF23_16; **14:** 2497318..2509976, 12659, 51.56, ORF23_326-ORF23_338; **15:** 2774280..2789036, 14757, 36.42, ORF25_143-ORF25_155; **16:** 2981228..3042369, 61142, 47.47, ORF29_1-ORF30_15; **17:** 3053731..3064085, 10355, 38.48, ORF30_24-ORF30_35; **18:** 3064394..3102497, 38104, 51.14, ORF30_36-ORF30_88; **19:** 3085955..3103008, 17054, 47.44, ORF30_61-ORF30_89; **20:** 3115857..3128027, 12171, 42.73, ORF31_2-ORF32_8; **21:** 3315841..3366201, 50361, 45.74, ORF34_136-ORF37_5; **22:** 3448512..3471101, 22590, 46.04, ORF39_2-ORF44_4; **23:** 3626872..3636320, 9449, 41.58, ORF58_4-ORF58_14; **24:** 3744081..3754030, 9950, 51.86, ORF67_72-ORF67_80.

Figures 1A–D presents the GI, T6SS, and prophage regions harbored by the strains. Strain 302-73 harbors 12 GI regions, 1 T6SS region, and 4 prophage regions, with the GC content of 37.31-54.02% and the size of 8729–14,7461 bp (Figure 1A). Strain LS1 harbors 20 pathogenicity-associated regions comprising 15 GI-like regions, 5 prophages, and 1 T6SS region, with the GC content of 36.75–57.02% and the size of 8203–74,732 bp (Figure 1B). Strain NCTC10360 harbors 12 regions (1 T6SS region, 2 prophage regions, and 9 GI regions) with the GC content of 34.23-51.75% and the size of 9960-81,386 bp (Figure 1C). Finally, strain GN7 harbors 25 regions (18 GI regions, 1 T6SS region, and 6 prophage regions), with the GC content of 36.42-54.15% and the size of 9449-61,142bp (Figure 1D).

### Putative Virulence Factor

Out of 134 unique PVFs identified in all the strains, strain 302-73 harbored 98 PVFs; strain GN7 harbored 107 PVFs; strain LS1 harbored 97 PVFs; and strain NCTC10360 harbored 90 PVFs. Detail information on the ORFs, amino acid length of predicted encoded proteins, *Ha*-values, and functional description of the homologous or orthologous predicted genes are provided in the Supplementary Data Sheets S1–S4.

**FIGURE 2:**
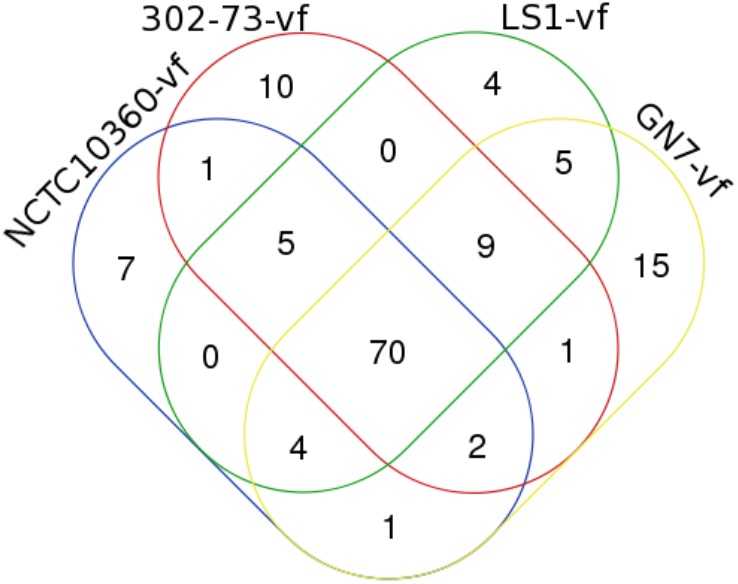
A Venn diagram of virulence factors in four *Plesiomonas shigelloides* strains.

The distribution of VFs among the strains is presented in Figure 2, with the VF summary given in Table 2. Of these, 70 (52.24% of 134) VFs were common to all the strains. The common VFs were mostly flagellum genes (27.14% of 70; *flgF, flgG, fliM, flgC, fliE, flgB, fliA, fliP, flgH, fleN, fleQ, flhB, fliG, flgC, fliI, flH, fliN, fliQ*, and *flgI*) related to those found in *Pseudomonas, Yersinia*, and *Legionella*. Other VFs common to all the strains included genes homologous or orthologous to *Salmonella mgtB, rpoS, csgE, pipD*, and *phoQ*; *Shigella orf9, fur, gspD, gspE, gspF, gspG, gspJ, pykF, csgF*, and *gspK*; *Legionella iraB, relA, letA, htpB, sodB, katB, ccmC*, and *motC*; *Campylobacter kpsF* and *gmhA*; *Pseudomonas pilT, algU, pilB, xcpA/pilD, waaF*, and *pilU*; *Mycobacterium mas, icl, glnA1*, and *panC*; *Haemophilus rfaD* and *rfaE*; *Yersinia galE*; *Vibrio vasA, vasE, tagD*, and *vasI*; *Listeria ClpP*; *Escherichia ompA, orf7, orf47, orf48*, and *fepC*; and *Staphylococcus cap8O* and *cap8E.* Five VFs (3.73% of 134) similar to genes found in *Pseudomonas* (*pilA, fliR*, and *waaC*), *Vibrio* (*vasH*), and *Legionella* (*feoB*) were only found in strains 302-73, LS1, and NCTC10360. Similarly, VF genes homologous or orthologous to *Escherichia orf45* and *Bordetella bplL* were identified in strains 302-73, GN7, and NCTC10360. Further, strains GN7, LS1, and NCTC10360 shared 2.99% (4 of 134) VFs, including *Escherichia StcE, Bordetella bplF, Salmonella sodCI*, and *Helicobacter neuA/flmD* homo-orthologs. VF gene subsets (6.72%; 9 of 134) identified only in strains 302-73, GN7 and LS1 were related to *Legionella CsrA*; *Vibrio vasC* and *nanE*; *Salmonella orf319, phoP*, and *ssb*; *Streptococcus cps4I*; *Helicobacter kdtB*; and *Listeria ClpC*. Virulence genes that were specifically found only in strains 302-73 and NCTC10360 were homologous to *Salmonella csgG*; those specific for strains GN7 and NCTC10360 were homologous to *Shigella orf34*; and those specific for strains 302-73 and GN7 were homologous to *Pseudomonas waaA*. VF genes homologous to *Mycobacterium panD, Vibrio nanA, Staphylococcus icaA, Helicobacter neuB*, and *Staphylococcus icaB* were identified only in strains GN7 and LS1.

**Table 2:** Summary of VFs in *P. shigelloides* strains.

Names	Total	% of 134	VFs with pathogens with related genes
302-73, GN7, LS1,NCTC10360	70	52.24	*Pseudomonas flgF, Salmonella mgtB, Shigella orf9, Legionella iraB, Legionella relA, Salmonella rpoS, Shigella gspF, Shigella gspK, Campylobacter kpsF, Pseudomonas flgG, Pseudomonas pilT, Escherichia orf48, Mycobacterium mas, Salmonella csgF, Haemophilus rfaE, Shigella gspG, Yersinia galE, Pseudomonas fliM, Salmonella fur, Legionella flgC, Campylobacter gmhA, Pseudomonas pilB, Pseudomonas fliE, Vibrio vasA, Mycobacterium glnA1, Pseudomonas xcpA/pilD, Shigella gspD, Mycobacterium panC, Yersinia flgB, Salmonella phoQ Listeria ClpP, Legionella katB Pseudomonas algU, Escherichia orf47, Legionella sodB, Shigella gspE, Salmonella csgE, Legionella fliA, Pseudomonas fliP, Vibrio vasE, Vibrio tagD, Haemophilus rfaD, Pseudomonas flgH Escherichia fepC, Pseudomonas fleN, Staphylococcus cap8O, Pseudomonas fleQ, Pseudomonas flhB, Pseudomonas pilU, Shigella gspJ, Escherichia ompA, Pseudomonas waaF, Staphylococcus cap8E, Pseudomonas fliG, Pseudomonas flgC, Pseudomonas fliI, Legionella motC, Salmonella pipD, Mycobacterium icl, Pseudomonas flH, Legionella ccmC, Salmonella pykF, Pseudomonas fliN, Vibrio vasI Pseudomonas fliQ, Legionella htpB, Legionella letA, Pseudomonas flgI, Escherichia orf7, Legionella pilC*
302-73, LS1 NCTC10360	5	3.73	*Pseudomonas waaC, Vibrio vasH Pseudomonas fliR, Legionella feoB, Pseudomonas pilA*
302-73, GN7, NCTC10360	2	1.49	*Escherichia orf45, Bordetella bplL*
GN7, LS1, NCTC10360	4	2.99	*Escherichia stcE, Bordetella bplF, Salmonella sodCI, Helicobacter neuA/flmD*,
302-73, GN7, LS1	9	6.72	*Legionella csrA, Vibrio nanE, Salmonella orf319, Streptococcus cps4I, Salmonella ssb Vibrio vasC, Salmonella phoP, Helicobacter kdtB, Listeria ClpC*
302-73, NCTC10360	1	0.75	*Salmonella csgG*
GN7, NCTC10360	1	0.75	*Shigella orf34*
302-73-vf GN7	1	0.75	*Pseudomonas waaA*
GN7-vf LS1	5	3.73	*Mycobacterium panD, Vibrio nanA, Staphylococcus icaA, Helicobacter neuB, Staphylococcus icaB*
NCTC10360	7	5.22	*Vibrio VC1772, copR, Salmonella ssb, Pseudomonas waaA, Bordetella bplG, Streptococcus cps4I, Helicobacter kdtB*
302-73	10	7.46	*Brucella cgs, Campylobacter hddC, Streptococcus neuC, Vibrio nanA, Staphylococcus cap8G, Staphylococcus cap8F, Brucella per, Escherichia stcE, Streptococcus neuB, Campylobacter ptmA*
LS1	4	2.99	*Vibrio hsdR, Salmonella rhuM, Vibrio VC1809, Vibrio hsdM*
GN7	15	11.19	*Escherichia z1204, Yersinia gmd, Yersinia manC, Escherichia intl, Escherichia ibeB, Pseudomonas fliR, Escherichia z1203, Escherichia orf72, Escherichia z1216, Escherichia orf51, Yersinia manB, Vibrio radC, Legionella feoB, Escherichia orf86, Salmonella copR*


The number of strain-specific VFs ranged from 4 to 15 (out of 134) in the four *Plesiomonas* strains. Specific to strain NCTC10360 were VFs that shared homo/orthology with *Vibrio VC1772, copR, Salmonella ssb, Pseudomonas waaA, Bordetella bplG, Streptococcus cps4I*, and *Helicobacter kdtB*. VFs related to *Brucella cgs* and *per, Campylobacter hddC, Streptococcus neuC, Vibrio nanA, Staphylococcus cap8G* and *cap8F, Escherichia StcE, Streptococcus neuB*, and *Campylobacter ptmA* were specific to strain 302-73. Exclusive to strain LS1 were ORF homologous/orthologous to *Vibrio hsdR, VC1809*, and *hsdM*; and *Salmonella rhuM*. In addition, 15 virulence genes related to *Escherichia z1204, intl, ibeB, z1203, orf72, z1216, orf51*, and *orf86*; *Pseudomonas fliR*; *Yersinia gmd, manC*, and *manB*; *Vibrio radC*; *Legionella feoB*; and *Salmonella copR* were only found in strain GN7.

The distribution of IUSRVTs among the strains is presented in Supplementary Figure S1. Of these, 9 IUSRVTs were common to all the strains. The common IUSRVTs were multispecies ubiquinol-cytochrome c reductase iron-sulfur subunit, multispecies iron donor protein *CyaY*, multispecies iron-sulfur cluster insertion protein *ErpA*, multispecies succinate dehydrogenase/fumarate reductase iron-sulfur subunit, zinc/iron-chelating domain-containing protein, iron export ABC transporter permease subunit *FetB*, iron-sulfur cluster repair di-iron protein, ferrous iron transport protein B, multispecies iron-sulfur cluster assembly protein *IscA*. Two IUSRVTs namely multispecies zinc/iron-chelating domain-containing protein and iron transporter *FeoA* were only found in strains 302-73, LS1, and NCTC10360. Similarly, the iron ABC transporter permease was identified in strains 302-73, GN7, and NCTC10360. Further, strains 302-73, GN7 and LS1 shared 2 IUSRVTs, including multispecies succinate dehydrogenase iron-sulfur subunit and multispecies iron-sulfur cluster scaffold-like protein. IUSRVTs that were specifically found only in strains 302-73 and LS1 were iron(III) ABC transporter ATP-binding protein. IUSRVTs exclusive to strain NCTC10360, GN7 and 302-73 was succinate dehydrogenase iron-sulfur subunit, multispecies iron transporter *FeoA* and iron(III) ABC transporter permease respectively.

The distribution of HUSRVTs among the *P. shigelloides* strains are summarized in Supplementary Figure S2. The various HUSRVTs common to all the strains ranged from heme ABC transporter ATP-binding protein, heme exporter protein *CcmB*, heme utilization protein *HutZ*, to heme ABC exporter ATP-binding protein *CcmA.* Strains GN7, LS1, and NCTC10360 encoded putative heme utilization radical SAM enzyme. Strains 302-73, GN7, and LS1 encoded biliverdin-producing heme oxygenase and multispecies heme exporter protein *CcmD*. Strains 302-73 and NCTC10360 possessed heme exporter protein *CcmC*. Specific to strain NCTC10360 were genes encoding heme exporter protein *CcmD*. Other HUSRVTs were exclusively shared by strains GN7 and LS1 (heme ABC transporter permease and heme peroxidase).

### AR Elements

**FIGURE 3:**
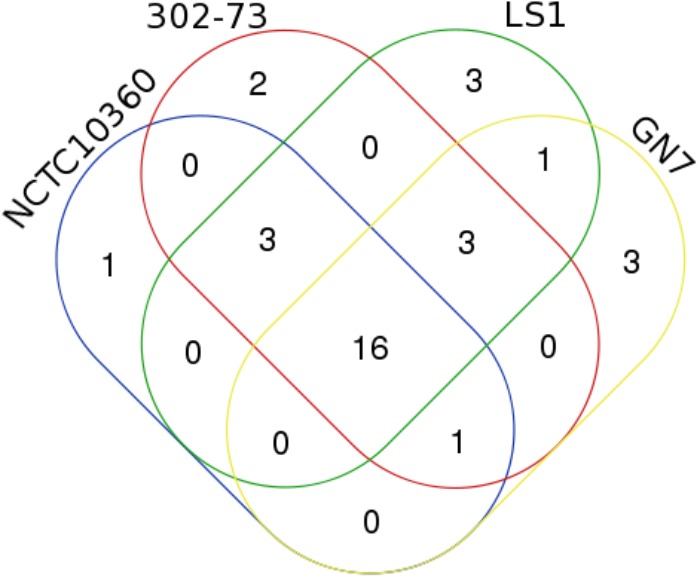
A Venn diagram of ARs in four *Plesiomonas shigelloides* strains.

About 20 distinctive AR elements were identified in strains 302-73 (25 genes), GN7 (24 genes), LS1 (26 genes), and NCTC10360 (21 genes) (Figure 3). Overall, 33 unique AR elements were identified in all the strains, as summarized in Table 3. Of the 33 AR elements, 16 (*emrD, catB5, mexF, mexB, mexW, vansD, blaB4, tcmA, macB, ksgA, pbp1A, bcrA, tolC, tet34, mdtK*, and *pbp2*) were common to all the strains. Additional three AR elements were found in strains 302-73, LS1, and NCTC10360 (*bcr, vanrB, dfra26*); three in strains 302-73, GN7, and LS1 (*acrB, bacA*, and *acrA*); and one in strains 302-73, GN7, and NCTC10360 (*ampE*). Undecaprenyl-diphosphatase gene was specifically found in strain NCTC10360, and *catA1* and *tetC* were specifically found in strain 302-73. Other AR elements were exclusively harbored by strain LS1 (*qnrB, ampE*, and *ble*) and GN7 (*oprM, smeF*, and *pbpD*).

**Table 3:** Summary of ARs in *P. shigelloides* strains.

Strain (s)	Total	% of 33	AR genes
302-73, GN7, LS1, NCTC10360	16	48.48	*emrD, catB,5 mexF, mexB, mexW, vansD, blaB4, tcmA, macB, ksgA, pbp1A, bcrA, tolC, tet34, mdtK, pbp2*
302-73 LS1 NCTC10360	3	9.09	*Bcr, vanrB, dfrA26*
302-73, GN7, NCTC10360	1	3.03	*ampE*
302-73, GN7, LS1	3	9.09	*acrB, bacA, acrA*
GN7, LS1	1	3.03	*tetA*
NCTC10360	1	3.03	*Undecaprenyl-diphosphatase*
302-73	2	6.06	*catA1, tetC*
LS1	3	9.09	*qnrB, ampE protein, ble*
GN7	3	9.09	*oprM, smeF, pbpD*


### Antibiotic Resistance Island

**FIGURE 4:**
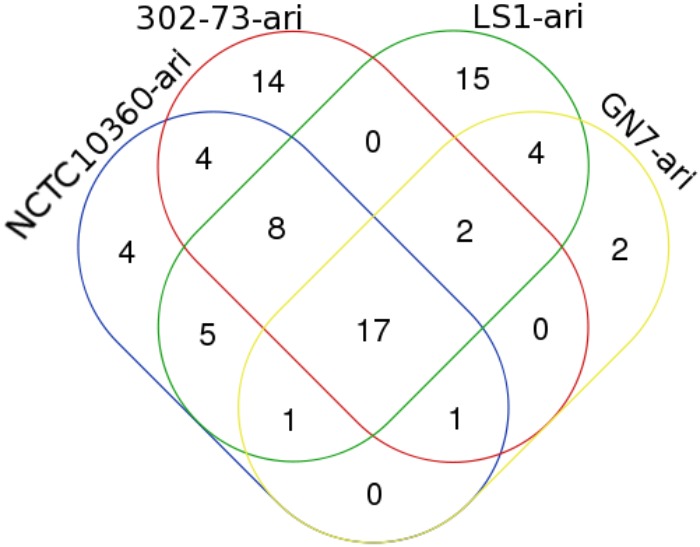
A Venn diagram of ARIs in four *Plesiomonas shigelloides* strains.

Figure 4 shows the distribution of ARIs among the strains. The various ARIs common to the strains were related to genes from *Pseudomonas aeruginosa* (4/17), *Acinetobacter baumannii* (10/17), *Pseudomonas mirabilis* (1/17), *Staphylococcus aureus* (1/17), and *Shigella flexneri* 2a (1/17) (Table 4). These included genes encoding putative heme exporter protein C, putative metal-transporting P-type ATPase, FklB, IntI1 integrase, ArsC, WecB, AspS, CcmF, Gpi, IlvE, lipoprotein signal peptidase, a putative amino acid transport protein, type 1 capsule synthesis protein, GalU, Gne1, MviN, and ATP-binding protein FecE. ARIs specific to strain NCTC10360 encoded a transcriptional regulator, regulatory protein, putative cytochrome C maturation protein B, and PsaC. Most ARIs solely found in strain 302-73 showed homology/orthology with genes encoding QhbA, LgaF, LgaD, LgaC, FnlA, FnlB, FnlC, AciD, LgaA, LgaB, chloramphenicol acetyltransferase, and QhbB from *A. baumannii*; and ATP-dependent endonuclease and potassium-transporting ATPase from *P. mirabilis* and *S. aureus*, respectively. Fifteen ARIs closely related to *A. baumannii* transposase, IS, transposase of IS26, and PsaC; *K. pneumoniae* transposase (plasmid) and Orf3 (plasmid); *P. mirabilis* IS26 transposase, DfrA1 protein, GroEL, hypothetical protein, and membrane partial protein; *S. flexneri* 2a putative protein Shf, putative transcriptional regulator, and putative anaerobic decarboxylate transporter; and *S. aureus* bleomycin resistance protein were unique to strain LS1. Sequences similar to ones encoding Pgm and type I restriction-modification system DNA methylase from *A. baumannii* and *S. aureus*, respectively, were only identified in strain GN7. Other ARIs shared by either two or three strains included ones encoding TrxB, putative aldehyde dehydrogenase, Wzc, *S. aureus* hypothetical protein, *S. aureus* unknown protein, diaminobutyric acid aminotransferase, Gna, ArmR, putative heme exporter protein A, putative anaerobic decarboxylate transporter, PsaF, putative cytochrome C maturation protein B, ArsB, *P. mirabilis* membrane partial protein, ItrA2, GroEL, Gdr, PsaB, PsaA, integrase, Gne2, *A. baumannii* repressor protein, *P. mirabilis* transposase and PecM, TetA(A), *P. mirabilis* transcriptional regulator and regulatory protein, putative cytochrome C maturation protein B, and PsaC.

**Table 4:** Summary of antibiotic resistance islands in *P. shigelloides* strains.

Strain (s)	Total	% of 77	ARI genes
302-73, GN7, LS1, NCTC10360	17	22.08	Putative heme exporter protein C *P. aeruginosa*,, putative metal-transporting P-type ATPase *P. aeruginosa, FklB A. baumannii*, IntI1 integrase *P. mirabilis, ArsC A. baumannii, WecB A. baumannii*, AspS *A. baumannii, CcmF Pseudomonas aeruginosa, Gpi A. baumannii*, IlvE *A. baumannii*, lipoprotein signal peptidase *A. baumannii*, putative amino acid transport protein *P. aeruginosa*, type 1 capsule synthesis gene *S. aureus, GalU A. baumannii, Gne1 A. baumannii, MviN A. baumannii*, ATP-binding protein *FecE S. flexneri* 2a
302-73, LS1, NCTC10360	8	10.39	*TrxB A. baumannii*, putative aldehyde dehydrogenase *P. aeruginosa, Wzc A. baumannii*, hypothetical protein *S. aureus*, diaminobutyric acid aminotransferase *P. aeruginosa, Gna A. baumannii, ArmR P. aeruginosa*, putative heme exporter protein A *P. aeruginosa*
302-73, GN7, NCTC10360	1	1.30	putative anaerobic decarboxylate transporter *Shigella flexneri* 2a
GN7, LS1, NCTC10360	1	1.30	*PsaF A. baumannii*
302-73, GN7, LS1	2	2.60	Putative cytochrome c maturation protein B *P. aeruginosa, ArsB A. baumannii*
302-73, NCTC10360	4	5.19	Membrane protein, partial *P. mirabilis*, ItrA2 *A. baumannii, GroEL P. mirabilis, Gdr A. baumannii*
LS1, NCTC10360	5	6.49	Unknown *S. aureus, PsaB A. baumannii, PsaA A. baumannii*, integrase *P. mirabilis, Gne2 A. baumannii*
GN7, LS1	4	5.19	Repressor protein *A. baumannii*, transposase *P. mirabilis, PecM P. mirabilis, tetA(A) A. baumannii*
NCTC10360	4	5.19	Transcriptional regulator proteus mirabilis regulatory protein *P. mirabilis*, putative cytochrome C maturation protein B *P. aeruginosa, PsaC A. baumannii*,
302-73	14	18.18	*QhbA A. baumannii, LgaF A. baumannii, LgaD A. baumannii, LgaC A. baumannii*, FnlC *A. baumannii*, FnlB *A. baumannii*, AciD *A. baumannii, LgaA A. baumannii, LgaB A. baumannii*, ATP-dependent endonuclease *P. mirabilis*, FnlA *A. baumannii*, chloramphenicol acetyltransferase *A. baumannii*, potassium-transporting ATPase, *S. aureus, QhbB A. baumannii*
LS1	15		Transposase *A. baumannii, DfrA1 P. mirabilis, PsaC A. baumannii*, putative protein Shf *S. flexneri* 2a, Orf3 (plasmid) *K. pneumoniae*, putative transcriptional regulator *S. flexneri* 2a, insertion sequence *A. baumannii, GroEL P. mirabilis*, transposase (plasmid) *K. pneumoniae*, transposase of *IS26 A. baumannii*, IS26 transposase *P. mirabilis*, hypothetical protein *P. mirabilis*, bleomycin resistance protein(BRP) *S. aureus*, putative anaerobic decarboxylate transporter *S. flexneri* 2a, membrane protein partial *P. mirabilis*
GN7	2		*Pgm A. baumannii*, type I restriction-modification system DNA methylase *S. aureus*


### Pathogenicity Islands

**FIGURE 5:**
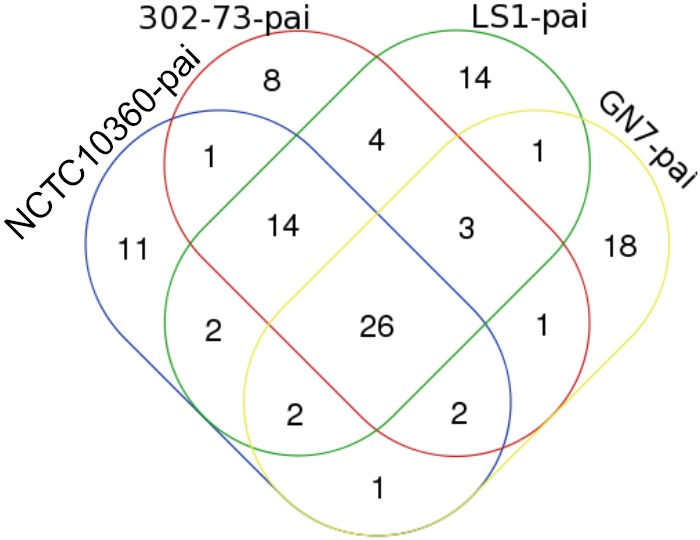
A Venn diagram of PAIs in four *Plesiomonas shigelloides* strains.

Pathogenicity islands identified in the *P. shigelloides* strains are summarized in Table 5 and Figure 5 shows the distribution of PAIs among the strains. The various PAIs common to all the strains ranged from unnamed or hypothetical proteins related to *Escherichia coli, Aeromonas hydrophila, Edwardsiella tarda*, and *Yersinia pestis*. Examples include PAIs encoding Orf77, Orf59, Epd, RnhA, Orf71, and Orf38 from *Photorhabdus luminescens*; VasA, Vask, Clpb protein, and VgrG-3 protein from *A. hydrophila*; DNA-binding protein StpA (plasmid) (*Klebsiella pneumoniae* subsp. *pneumoniae* kp13); Inti2, GspD, GspF, putative cadaverin decarboxylase GspE, GspK, GspG, and GspJ (*E. coli*); ATP-binding protein FecE (*S. flexneri* 2a), and AruF (*Y. pestis*).

**Table 5:** Summary of PAIs in *P. shigelloides* strains.

Names	Total	% of 108	Pai and possible donor (origin)
302-73, GN7, NCTC10360, LS1	26	24.07	Hypothetical protein *Escherichia coli*, hypothetical protein *A. hydrophila, Orf77 Photorhabdus luminescens, VgrG-3* protein *A. hydrophila*, DNA-binding protein StpA (plasmid) *K. pneumoniae* subsp. Pneumoniae kp13, *Vask A. hydrophila, GspE E. coli*, Unknown *Photorhabdus luminescens*, ATP-binding protein fecE *S. flexneri* 2a, *Orf59 Photorhabdus luminescens, Inti2 E. coli*, Hypothetical protein *Edwardsiella tarda*, Unnamed protein product *Y. pestis, GspD E. coli, GspK E. coli, Epd Photorhabdus luminescens, Orf71 Photorhabdus luminescens, GspF E. coli*, Putative cadaverin decarboxylase *E. coli, Orf38 Photorhabdus luminescens, VasA A. hydrophila, GspJ E. coli, RnhA Photorhabdus luminescens*, Clpb protein *A. hydrophila*, GspG *E. coli*, AruF *Y. pestis*,
302-73, LS1, NCTC10360	14	12.96	*OmpA/motB Delftia tsuruhatensis, Orf33 Photorhabdus luminescens*, Ubiquinone biosynthesis o-methyl-transferase Delftia acidovorans DNA gyrase a subunit *Delftia acidovorans VasF A. hydrophila*, Putative lysil-TRNA synthetase *lysU E. coli, VasH A. hydrophila, Orf17 Photorhabdus luminescens*, putative phage-related protein *Yersinia enterocolitica*, putative oxidoreductase *Yersinia pseudotuberculosis*, KpsU protein *E. coli*, Putative DNA binding protein with DNA-dependent ATPase activity *Y. pseudotuberculosis, Pgk Photorhabdus luminescens, GloB Photorhabdus luminescens*
302-73, GN7, NCTC10360	2	1.85	ToxR-activated gene D protein *Vibrio cholerae, QueA Pseudomonas syringae*
GN7, LS1, NCTC10360	2	1.85	Arub *Y. pestis*, putative anaerobic decarboxylate transporter *S. flexneri 2a*
302-73, GN7, LS1	3	2.78	Putative prepilin peptidase A *E. coli, VgrG-2* protein *A. hydrophila*, Hcp-2 hemolysin-coregulated protein *A. hydrophila*
302-73, NCTC10360	1	0.93	3-oxoacyl-acyl carrier protein synthase ii *Pseudomonas savastanoi*
NCTC10360, LS1	2	1.85	*AruD Y. pestis*, Polysialic capsule transport protein *E. coli*
NCTC10360, GN7	1	0.93	Unknown *S. flexneri* 2a
302-73, LS1	4	3.70	*TktA Photorhabdus luminescens*, conserved hypothetical protein *Streptomyces lividans*, putative lysin/cadaverin transporter *E. coli, AruC Y. pestis*
302-73, GN7	1	0.93	Putative cadaverin decarboxylase *E. coli*
GN7, LS1	1	0.93	*Hmsh Y. pestis*
NCTC10360	11	10.19	*Orf58 Photorhabdus luminescens, KtA Photorhabdus luminescens*, conserved hypothetical protein *S. lividans*, nucleotidyltransferase *Pseudomonas savastanoi, RepC* partial (plasmid) *K. pneumoniae*, putative prepilin peptidase A *E. coli, Parf* (plasmid) *E. coli*, putative phage-related protein *Yersinia enterocolitica*, ToxR-activated gene A protein Vibrio cholerae, Putative cadaverin transporter *E. coli* Unnamed protein product
302-73	8	7.41	Phage integrase *E. coli, TnpB S. flexneri* 5a, ToxR-activated gene A protein *V. cholerae*, DNA *Q Photorhabdus luminescens*, Polysialic capsule transport protein *E. coli, AruD Y. pestis*, putative anaerobic decarboxylate transporter *S. flexneri* 2a, nucleotidyltransferase/DNA polymerase *Pseudomonas savastanoi, AruB Y. pestis*
LS1	14	12.96	DNA Q *Photorhabdus luminescens*, hypothetical protein *E. coli*, dihydrofolate reductase *Staphylococcus epidermidis, HmsR Y. pestis*, nucleotidyltransferase/DNA polymerase *Pseudomonas savastanoi*, hypothetical protein *A. hydrophila*, ToxR-activated gene D protein *V. cholera*, resolvase (plasmid) *k. pneumoniae* subsp. *Pneumoniae* kp13, IS1 repressor protein insA (plasmid) *E. coli*, ToxR-activated gene A protein *V. cholera, HmsF Y. pestis QueA, P. syringae*, Integrase V. *cholerae, GspE E. coli*
GN7	18	16.67	Z1204 protein *E. coli*, putative hsdm-like n-methyl transferase *Y. pseudotuberculosis*, replicative DNA helicase DNA B *Y. pseudotuberculosis*, putative cadaverin decarboxylase *E. coli*, Orf48 *E. coli, VgrG-3* protein/*vgrG-2* protein *A. hydrophila, TktA Photorhabdus luminescens, pgK Photorhabdus luminescens*, mobilization protein C (plasmid) *Pasteurella aerogenes*, Int/cp4-like integrase *E. coli*, putative oxidoreductase *Y. pseudotuberculosi*s, Integrase *V. cholerae*, cadaverin transporter *E. coli, EvfF E. coli*, RS218 *vasF A. hydrophila*, stability protein *stbE* (plasmid) *E. coli*, hypothetical protein *A. hydrophila, KpsU* protein *E. coli*


Strains 302-73, GN7, and NCTC10360 encoded ToxR-activated gene D protein and QueA similar to the respective proteins from *V. cholerae* and *Pseudomonas syringae*, respectively. Strains 302-73, GN7, and LS1 encoded proteins similar to putative prepilin peptidase A from *E. coli*, and VgrG-2 protein and Hcp-2 hemolysin-coregulated protein from *A. hydrophila*. Specific to strain NCTC10360 were genes encoding Orf58 and KtA related to proteins from *Photorhabdus luminescens*, conserved hypothetical protein (*Streptomyces lividans*), nucleotidyltransferase (*Pseudomonas savastanoi*), RepC partial plasmid (*K. pneumoniae*), putative prepilin peptidase A (*E. coli*), ParF plasmid protein (*E. coli*), putative phage-related protein (*Yersinia enterocolitica*), and ToxR-activated gene A protein (*V. cholerae*). PAIs solely harbored by the strain 302-73 encoded phage integrase and polysialic capsule transport protein (*E. coli*); TnpB (*S. flexneri* 5a); ToxR-activated gene A protein (*V. cholerae*); DNA Q protein (*Photorhabdus luminescens*); putative anaerobic decarboxylate transporter (*S. flexneri* 2a); nucleotidyltransferase (*Pseudomonas savastanoi*); and AruB and AruD (*Y. pestis*). DNA Q protein (*Photorhabdus luminescens*), hypothetical proteins (*E. coli* and *A. hydrophila*), dihydrofolate reductase (*Staphylococcus epidermidis*), HmsF and HmsR (*Y. pestis*), DNA polymerase (*Pseudomonas savastanoi*); integrase, ToxR-activated gene A and D proteins (*V. cholerae*); resolvase (plasmid) (*K. pneumoniae*), IS1 repressor protein InsA (plasmid) and GspE (*E. coli*); and QueA (*P. syringae*) were putative PAIs specific to strain LS1. Strain GN7 was the only one harboring PAIs encoding Z1204 protein, stability protein StbE (plasmid), EvfF, Int/cp4-like integrase, putative cadaverin decarboxylase, cadaverin transporter, KpsU protein, and Orf48 (*E. coli*); putative Hsdm-like *N*-methyl transferase, putative oxidoreductase, and replicative DNA helicase DNA B (*Yersinia pseudotuberculosis*); RS218, VasF, hypothetical protein, and VgrG-3 protein/VgrG-2 protein (*A. hydrophila*); Pgk and TktA (*Photorhabdus luminescens*); mobilization protein C plasmid (*Pasteurella aerogenes*); and integrase (*V. cholerae*).

### IS Elements

**FIGURE 6:**
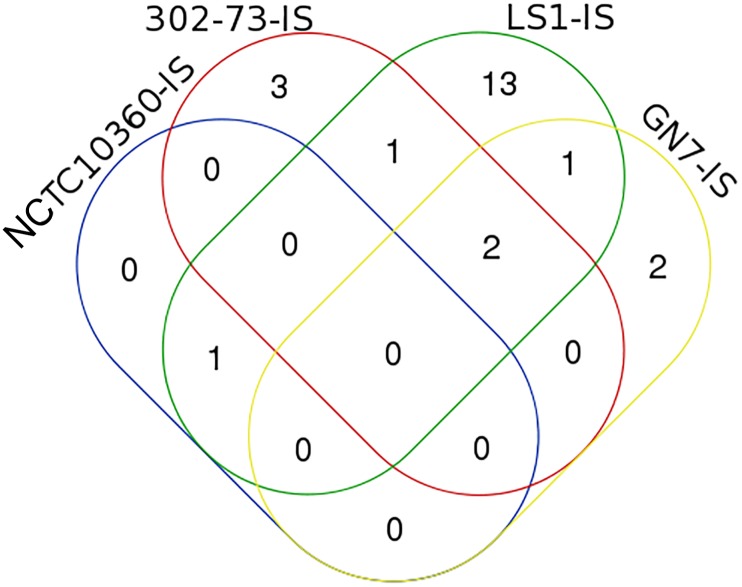
A Venn diagram of ISs in four *Plesiomonas shigelloides* strains. The identities of elements shared or specific to the sub-region of the Venn diagram is presented against it in parenthesis as follows: 302-73, GN7, and LS1 (ISEhe3_PEP, and ISAbo1_PEP3), NCTC10360 and LS1 (ISSm4_PEP2), 302-73 and LS1 (IS1N_PEP), GN7 and LS1 (ISIba1_PEP3), 302-73 (ISEc16_PEP3, IS3F_PEP, ISAs1_PEP), GN7 (ISErsp1_PEP, ISIba2_PEP), LS1 (ISShes11_PEP5, ISEic1_PEP3, IS15DII_PEP, ISIba2_PEP3 ISSag9_PEP, ISStso1_PEP, ISStso1_PEP3, ISKpn25_PEP3, IS1381A_PEP3, IS1381A_PEP ISEic1_PEP ISSm4_PEP, ISEhe3_PEP3).

The IS elements identified in the four *P. shigelloides* strains are presented in Table 6. The IS elements were shared by the strains or specific to a given strain, as follows: ISEhe3_PEP, and ISAbo1_PEP3 were identified in strains 302-73, GN7, and LS1; ISSm4_PEP2 was identified in strains NCTC10360 and LS1; IS1N_PEP was identified in strains 302-73 and LS1; ISIba1_PEP3 was identified in strains GN7 and LS1; ISEc16_PEP3, IS3F_PEP, and ISAs1_PEP were only identified in strain 302-73; ISErsp1_PEP and ISIba2_PEP were only identified in strain GN7; ISShes11_PEP5, ISEic1_PEP3, IS15DII_PEP, ISIba2_PEP3 ISSag9_PEP, ISStso1_PEP, ISStso1_PEP3, ISKpn25_PEP3, IS1381A_PEP3, IS1381A_PEP ISEic1_PEP ISSm4_PEP, and ISEhe3_PEP3 were only identified in strain LS1. The distribution of IS elements among the strains is depicted in Figure 6. LS1 harbored 56.52% (13) of all IS elements identified in the strains.

**Table 6:** Summary of ISs in *P. shigelloides* strains.

Strain (s)	Total	% of 23	IS
302-73,GN7, LS1	2	8.70	ISEhe3_PEP, ISAbo1_PEP3
NCTC10360, LS1	1	4.35	ISSm4_PEP2
302-73, LS1	1	4.35	IS1N_PEP
GN7, LS1	1	4.35	ISIba1_PEP3
302-73	3	13.04	ISEc16_PEP3, IS3F_PEP, ISAs1_PEP
LS1	13	56.52	ISShes11_PEP5, ISEic1_PEP3, IS15DII_PEP, ISIba2_PEP3 ISSag9_PEP, ISStso1_PEP, ISStso1_PEP3, ISKpn25_PEP3, IS1381A_PEP3, IS1381A_PEP ISEic1_PEP ISSm4_PEP, ISEhe3_PEP3
GN7	2	8.70	ISErsp1_PEP, ISIba2_PEP


### SS Elements

**FIGURE 7:**
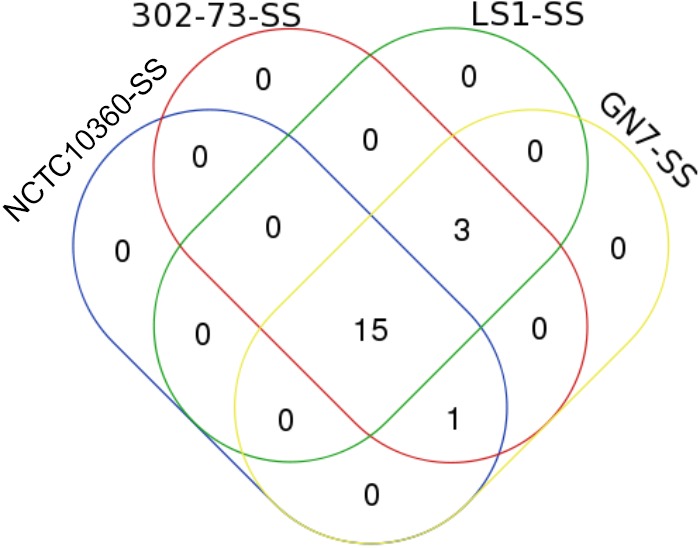
A Venn diagram of SSs in four *Plesiomonas shigelloides* strains.

Overall, 19 SS elements were identified in the strains (Table 7). The various SS elements included T6SS (encoding TssA, TssB, TssC, TssH, TssJ, TssK, TssF, TssL, TssM, TssG, and TssE); T3SS (encoding BscN and SctN), T4SE_29653378, and T6SE (encoding ABG57151, ABG57133, and ABG57132). The distribution of SS elements among the strains is shown in Figure 7; 78.95% of all SS elements (15 of 19) were harbored by all the strains, while 15.79% of all SS elements (3) were exclusive to 302-73, GN7, and LS1 strains.

**Table 7:** Summary of SSs in *P. shigelloides* strains.

Strain (s)	Total	% of 19	SS
NCTC10360, 302-73, GN7, LS1	15	78.95	T6SS_50122359_TssH, T3SS_BscN, T6SS_50122367_TssC, T6SS_50122356_TssA, T6SS_50122361_TssK, T6SS_50122362_TssJ, T6SS_50122365_TssF, T3SS_SctN, T6SS_50122360_TssL, T6SS_50122355_T ssM, T6SS_50122364_TssG, T6SS_50122366_TssE, T6SS_50122368_TssB, T6SS_108761289_TssH, T6SE_ABG57151
NCTC10360, 302-73, GN7	1	5.26	T4SE_29653378
302-73, GN7, LS1	3	15.79	T6SE_ABG57133, T6SS_50122354_TssA, T6SE_ABG57132


### CRISPR Elements

No CRISPR element was identified in strain GN7. Various features of CRISPR elements harbored by the other three *Plesiomonas* strains are detailed in Table 8. Strain NCTC10360 harbored two CRISPR elements (172 and 185 bp). The 172-bp element harbored three repeated sequences and two spacers, with a repeat length of 25 bp. The 185-bp element harbored four repeated sequences and three spacers, with a repeat length of 20 bp. Strain 302-73 harbored eight CRISPR elements with the total length ranging from 139 to 181 bp. Each of these elements harbored three repeated sequences and two spacers; the repeat length varied from 21 to 28 bp. Finally, in strain LS1, one CRISPR element (142 bp total length) was identified; it harbored three repeats and two spacers, with a repeat length of 22 bp. The structural and compositional details of CRISPRs identified in the *P. shigelloides* strains are presented in Supplementary Data Sheet S5.

**Table 8:** Detail characteristics of CRISPRs found in *P. shigelloides*.

S/N	Name	Locus range	Total length	No. of Repeats	No. of Spacers	Repeat length
	**NCTC10360**					
1	CRISPR1	634986 – 635157	172	3	2	25
2	CRISPR2	681979 – 682163	185	4	3	20
	**302-73**					
1	CRISPR_1	488056 – 488199	144	3	2	24
2	CRISPR_2	698058 – 698205	148	3	2	21
3	CRISPR_3	1317065 – 1317245	181	3	2	28
4	CRISPR_4	1317501 – 1317639	139	3	2	25
5	CRISPR_5	2287008 – 2287151	144	3	2	24
6	CRISPR_6	2497010 – 2497157	148	3	2	21
7	CRISPR_7	3116017 – 3116197	181	3	2	28
8	CRISPR_8	3116453 – 3116591	139	3	2	25
	**LS1**					
1	CRISPR_1	297760 – 297901	142	3	2	22


### Prophage Entities

The results of comparative phageomics of the four *P. shigelloides* strains are shown in Table 8. Strain NCTC10360 harbored two intact phages sized 21.9 kb and 53.8 kb, most similar to PHAGE_Entero_mEp235_NC_019708, PHAGE_Klebsi_phiKO2_NC_005857, PHAGE_Yersin_PY54_NC_005069, and PHAGE_Entero_lambda_NC_001416. Strain GN7 harbored two intact and one incomplete phage sized 36.2, 30.3, and 29.5 kb respectively. The GN7 phages were most similar to the following phage species: PHAGE_Flavob_1H_NC_031911, PHAGE_Shigel_Stx_NC_029120, PHAGE_Shigel_POCJ13_NC_025434, PHAGE_Salmon_Fels_2_NC_010463, and PHAGE_Salmon_Fels_2_NC_010463. Three incomplete phages and one questionable phage were identified in strain LS1. The only phage (31.1 kb) identified in strain 302-73 encoded tRNA and was similar to PHAGE_Salmon_RE_2010_NC_019488.

### Plasmid Elements

No plasmid element was identified in the four strains. Replicon information of the WGS revealed chromosomes.

## Discussion

One major problem of the conventional elucidation of pathogenesis mechanisms of *Plesiomonas* is the lack of a suitable experimental animal model. In the current study, comparative pathogenomics approach was used to detect virulence traits of the emerging pathogen that, until now, had been unresolved by traditional approaches. As an example, using an adult rabbit model, the ileal loop system, Sanyal et al. (1980) had established the diarrheagenic (enterotoxigenic) potential of *Plesiomonas* but did not model invasive pathogenesis. Further, pathogenomics studies revealed the presence of several genes associated with adherence and attachment to the villi of enterocytes (including thin aggregative fimbriae genes, such *csgE, csgF, csgG, gmhA, pilT*, and *pilU*) (Collinson et al., 1996; Sukupolvi et al., 1997; Römling et al., 1998; Comolli et al., 1999a; Bacon et al., 2001), characteristics necessary for diarrheagenic activity of pathogens. Pathogenomics not only support the findings of Sanyal et al. (1980), provided exhaustive genetic accessories that enable the bacterium with diarrheagenic potential.

Further, invasive models of *Plesiomonas* involving the eye (guinea pig or rabbit) and HeLa epithelial cells failed either to establish keratoconjunctivitis and internalized by the HeLa epithelial cells, respectively (Sanyal et al., 1980). Failed invasive investigations of *Plesiomonas* with HeLa cells were also reported by Pitarangsi et al. (1982), Holmberg and Farmer (1984), and Olsvik et al. (1990). Chowdhury et al. (1988) examined enteropathogenicity responses of adult rabbits orograstically dosed with *Plesiomonas* and reported that the rabbits were neither diarrheic nor deathward, except for soft acute inflammatory effects observed in the ileum mucosa. By contrast, homology shown by various *Plesiomonas* ORFs to genes linked to the invasiveness of other pathogens suggests its inherent propensity for invasive virulence. For instance, *Plesiomonas* homologs of invasive genes, such as *motC* (Heuner and Steinert, 2003), *clpP* (Gaillot et al., 2000; Gaillot et al., 2001), *flgC* (Heuner and Steinert, 2003), *fliA* (Dietrich et al., 2001), *flgB* (Young et al., 1999; Young et al., 2000), *ompA* (Prasadarao et al., 2002; Khan et al., 2003; Prasadarao et al., 2003; Sukumaran et al., 2003), LOS (*rfaE*) (Rao et al., 1999; Swords et al., 2000), *waaC* and *waaF* (Rocchetta et al., 1999; Lyczak et al., 2000), and *clpC* (Rouquette et al., 1996; Rouquette et al., 1998; Nair et al., 2000) have been shown to enhance the adhesion and invasion of different cells by other pathogens. This was further supported by various extraintestinal infections associated with *Plesiomonas*, including peritonitis (Alcañiz et al., 1995; Patel et al., 2016), cellulitis (Gopal and Burns, 1991; Jönsson et al., 1998), wound and foot infections (McCracken and Barkley, 1972; Herve et al., 2007; Pence, 2016), endophthalmitis (Butt et al., 1997; Mahrshmann and Lyons, 1998), and keratitis (Butt et al., 1997; Klatte et al., 2012). In addition, pneumonia (Schneider et al., 2009), migratory polyarthritis (Gupta, 1995), cholecystitis (Claesson et al., 1984; Kennedy et al., 1990), pyosalpinx (Roth et al., 2002), pseudo-appendicitis (Fischer et al., 1988), cholangitis and pancreatitis (Kennedy et al., 1990) are examples of infections that required invasive factor that have been reported caused by *Plesiomonas*. Further, Binns et al. (1984) showed that 31.3% of examined strains are invasive toward HeLa cells. Vitovec et al. (2001) showed that *Plesiomonas* monoinfection of neonatal mice resulted in long-term intestinal colonization and varied patho-histophysiological severity, ranging from an atrophic ileum, and ileal and colonic necrosis to colonic enterocytes. On the other hand, *Cryptosporidium parvum* co-infection resulted in dense ileal colonization, bacteremia, necrotizing inflammatory responses, diarrheic condition, and death. Of importance in this context is a *Plesiomonas* ORF homologous to *Escherichia ompA* identified in the current study. OmpA is associated with several virulence traits, including serum resistance, invasion, binding to C4b-binding protein, evasion of complement-mediated attack, and invasion of the microvascular endothelial cells in the brain via a ligand-receptor interaction (Prasadarao et al., 2002; Khan et al., 2003; Prasadarao et al., 2003; Sukumaran et al., 2003). These observations and comparative pathogenomics of *Plesiomonas* further attest to the probable invasive pathogenicity potential of this bacterium.

Other core pathogenic traits that remained unidentified or were only weakly supported by conventional experimentation include the cytotoxic and hemolytic potential of *Plesiomonas*. According to cell culture and animal model studies of *Plesiomonas* cytotoxicity, some strains produce extracellular cytotoxins with activities against Vero cells (Olsvik et al., 1990). Similarly, vacuolation and cytotoxigenic activities of plesiomonad strains recovered from different environments, including human/animals and aquatic bodies, in Vero cell monolayers were described by Ekman (2003) and Falcón et al. (2003), respectively. Although there is some evidence (as mentioned), other studies yield contradicting data. Vitovec et al. (2001) were unable to demonstrate either cytotoxic or hemolytic activity of *Plesiomonas* isolates during both, monoinfection and *C. parvum* co-infection in experimental models. Likewise, Pitarangsi et al. (1982) and Holmberg and Farmer (1984) concluded that the tested *Plesiomonas* strains are not cytotoxic in the animal model used. Further, the plesiomonad strains studied by Maluping et al. (2004) exhibited no cytotoxicity or hemolytic potential in any of the experimental models explored. Nonetheless, in the current study, we identified some plesiomonad ORFs homologous or orthologous to genes, such as *ssb*, known to mediate toxin secretion similar to *E. coli* hemolysin secretion (Wong et al., 1998; Marcus et al., 2000), and *xcpA*/*pilD*, known to facilitate the internalization and cytotoxicity in *P. aeruginosa* (Hahn, 1997; Comolli et al., 1999b). These suggest the cytotoxicity potential of *Plesiomonas*.

The LOS gene from *Haemophilus* that showed homology with a *Plesiomonas* ORF is linked to endotoxin and immunogenic functions, and its phosphoryl choline impacts the invasion via interaction with PAF receptor; and stimulates inflammatory signals, evasion of antigen-specific host defenses, and colonization of diverse microenvironments within the host (Rao et al., 1999; Erwin et al., 2000; Swords et al., 2000). A homologous ORF might provide *Plesiomonas* with a related pathogenic ability. Other genes related to toxin production and enteropathogenesis with homology to *Plesimonas* ORFs include *waaC* and *waaF*. These genes function in antiphagocytosis and resistance to serum killing (Rocchetta et al., 1999; Lyczak et al., 2000; Schroeder et al., 2002). Further, *Plesiomonas* possesses ORFs that are homologous to *Vibrio* PAI genes (*tagD, nanE*, and *nanA*). These Vibrio VFs are associated with cholera toxins and a putative protease-modulating VF in toxigenic *Vibrio* species (Karaolis et al., 1998; Jermyn and Boyd, 2002; Faruque and Mekalanos, 2003; Schmidt and Hensel, 2004). However, Herrington et al. (1987), using early traditional approaches, were unable to establish *Plesiomonas* enteropathogenesis in an animal model.

Virulence genes required for extraintestinal infections were identified in previous studies. Of these, serum resistance and antiphagocytosis factors, including *cap8E* and *cap8O* (Thakker et al., 1998; Cunnion et al., 2001; Luong and Lee, 2002; Cunnion et al., 2003), *algU* (Pier et al., 2001; Nivens et al., 2001; Song et al., 2003), and *bplF* (Harvill et al., 2000; Burns et al., 2003; Schaeffer et al., 2004) showed homology to some *Plesiomonas* ORFs.

Plesiomonad ORF homologs of such genes as *Staphylococcus icaA* and *icaB* suggest bacterial ability of intracellular survival within the host. These genes are associated with the synthesis of intercellular adhesin polysaccharide, proteins, and biofilm formation in *S. epidermidis* (Heilmann et al., 1996; McKenney et al., 1998; Cramton et al., 1999; Götz, 2002). Likewise, the presence of *Plesiomonas* gene homologous to *Salmonella rhuM* (*Salmonella* PAI 3) might suggest that it encodes a similar high-affinity magnesium transport system (*mgtCB*) required for intramacrophage survival fitness and virulence (Blanc-Potard and Groisman, 1997; Tao et al., 1998; Moncrief and Maguire, 1999). *Plesiomonas* harbor genes related to *katAB*, of which *katB* was shown to encode a catalase-peroxidase (Bandyopadhyay and Steinman, 1998, 2000; Bandyopadhyay et al., 2003), a stress protein essential for intracellular survival and transmission. A *Plesiomonas* gene homolog of *htpB* (encoding Hsp60 in *Legionella*) may function similarly to *htpB*, i.e., mediate complement-independent attachment to the host cells (Garduño et al., 1998; Swanson and Hammer, 2000). Other notable *Plesiomonas* VFs include ORFs that show homology to *Escherichia* enterobactin (*fepC*), *Legionella feoB*, and *Listeria clpC*. While *fepC* and *feoB* function in iron uptake in *Escherichia* (Cao et al., 2000; Sprencel et al., 2000) and *Legionella* (Robey and Cianciotto, 2002) respectively, ClpC mediates early escape of *Listeria* from macrophage phagosome, and adhesion and invasion (Rouquette et al., 1996; Rouquette et al., 1998; Nair et al., 2000). Further, *Plesiomonas* ORF related to *Salmonella fur* gene might enable iron regulation of gene expression in the bacterium, as reported for *Salmonella* (Guo et al., 1997; Lucas and Lee, 2000; Lejona et al., 2003).

The four *P. shigelloides* strains were replete with heme iron utilization systems (IUSRVTs/ HUSRVTs) early proposed as virulence mechanisms (Daskaleros et al., 1991; Santos et al., 1999; Henderson et al., 2001; Gonzalez-Rodriguez et al., 2007; Oldham et al., 2008; Rodríguez-Rodríguez and Santos, 2018). However, the roles of these iron acquisition systems in *P. shigelloides* pathogenesis require further transcriptomic validation in suitable models. While many authors found an association of iron uptake systems with virulence in some pathogens (Fisher et al., 2009; Watson et al., 2010), others found no direct relationship between them (Lindgren et al., 2011; Fletcher et al., 2018). Most importantly, the iron uptake systems (Feo Systems) are the essential cellular component for survival in pathogens, non-pathogens and commensal bacteria (Cartron et al., 2006; Lau et al., 2015). Some pathogens whose virulence depend on iron uptake systems include *Neisseria meningitidis* (Stojijkovic et al., 1995), *Salmonella* (Velayudhan et al., 2007; Nagy et al., 2014), *Campylobacter* (Naikare et al., 2006), *Helicobacter* (Velayudhan et al., 2000), *Legionella* (Robey and Cianciotto, 2002), *Haemophilus influenzae* (Morton et al., 2009), and others (Aranda et al., 2009; Cassat and Skaar, 2013).

The presence of ARIs and clinically relevant antibiotic (multi)resistance genes might limit the treatment and management options for the infections caused by *Plesiomonas*. This could have potentially serious consequences since *Plesiomonas* infections are not routinely diagnosed in the medical setting (Chen et al., 2013). Similar to many cases of antimicrobial resistance shown by various microorganisms, infections involving resistant *Plesiomonas* might be very difficult to manage, leading to extended hospitalization, high morbidity, and, ultimately, elevated mortality rates (Kain and Kelly, 1989b). Multi-resistance of zoonotic *Plesiomonas* could pose increased threat to human and veterinary health. This might further raise the associated possibility of dissemination and contact between human and animals, especially pets and livestock. *Plesiomonas* are by nature, β-lactamase producers (resistant to β-lactam drugs) (Meyers et al., 1976; Brenden et al., 1988; Fisher et al., 1988; Avison et al., 2000; Stock and Wiedemann, 2001a). Plesiomonad tetracycline resistance has been reported by Kain and Kelly (1989a); González et al. (1999), Wong et al. (2000), and Suthienkul et al. (2001). Co-resistance of plesiomonad strains to antibiotics, including ampicillin, amoxicillin/clavulanic acid, trimethoprim/sulfamethoxazole, chloramphenicol, streptomycin, tetracycline, erythromycin, sulphametoxazole, and neomycin has also been documented (Olsvik et al., 1990; González et al., 1999; Wong et al., 2000; Jun et al., 2011). Chloramphenicol-, co-trimoxazole-, erythromycin-, and rifampin-resistant *Plesiomonas* strains are frequently reported (Wong et al., 2000; Stock and Wiedemann, 2001b; González-Rey et al., 2004). Tobramycin- and gentamicin-resistant strains have also been documented (Clark et al., 1990). Tetracycline-resistant strains of *Plesiomonas* veterinary isolates are not uncommon. González-Rey et al. (2004) documented a multi-drug resistant *Plesiomonas* and tetracycline-resistant strain recovered from a Cuban dog and an environmental sample, respectively. Widespread tetracycline-resistant strains have also been noted among plesiomonad isolates from catfish ponds (DePaola et al., 1993). Further, aminoglycoside-resistant and penicillin-resistant *Plesiomonas* isolates were reported (Clark et al., 1990; Bravo-Fariñas et al., 1998; Avison et al., 2000; Stock and Wiedemann, 2001b). Yeh and Tsai (1991) have documented vancomycin-resistant strains of *Plesiomonas*. Furthermore, Jun et al. (2011) reported *Plesiomonas* resistance to amikacin, cefotaxime, cefepime, gentamicin, and ciprofloxacin. *Plesiomonas* inactivation of aminoglycoside antibiotics has been attributed to the production of specific enzymes, such as aminoglycoside-modifying enzymes (Shaw et al., 1993). Homology of *Plesiomonas* ORFs to heavy-metal resistance genes closely related to arsenic resistance in *Acinetobacter* spp. suggests the bacterium’s increasing ability to evade potential antiseptic treatment. Previously, Chao et al. (2016) reported cadmium-resistance in *Plesiomonas*, with a maximum tolerance concentration as high as 150 mg/L. Although Chao et al. (2016) concluded that this trait could be applicable in wastewater treatment, employed as an indicator of heavy metal pollution for eco-monitoring and environmental impact assessment, heavy metal resistance in *Plesiomonas* also suggests possible adverse implications for infectious cases.

The presence of *Plesiomonas* ORFs homologous to genes encoding different secretory and effector systems (T6SS, T3SS, T4SE, and T6SE) found in other pathogens might be indicative of a similar virulence function in the microorganism. For example, a *Yersinia* and *Salmonella* effector molecule that depolymerizes actin plays a similar role in tissue culture cells (Rosqvist et al., 1995). Secretory systems directly transport effector molecules from the cytoplasmic compartment to the cell surface to interact with and modify mammalian host cell proteins (Michiels et al., 1990). Other functional consequences of secretory systems in enteric and many other pathogens, such as *Yersinia* spp., *Shigella* spp., and *Salmonella* spp., include abolition of the immune cell function and engulfment ability, and macrophage protein modification (Rosqvist et al., 1990; Bliska et al., 1991; Cornelis, 1992); and enhanced pathogen entry into non-phagocytic cells (Galán, 1996; Ménard et al., 1996). Similar virulence traits are probably linked to the extraintestinal infections caused by *Plesiomonas*. Many genes harbored on or related to TSS elements, such as *gspD–J*, play roles in protecting pathogens against human complement activity and serum (Aiello et al., 2010; Johnson et al., 2016; Cianciotto and White, 2017; Waack et al., 2017). TSS elements are also involved in the mediation of cascades of secreted virulence effectors in gram-negative pathogens (Aiello et al., 2010; Johnson et al., 2016; Cianciotto and White, 2017).

Almost all *Plesiomonas* strains analyzed in the current study possessed PAIs homologous to those found in most enteropathogens. One of the PAIs, for instance, contained cadavarine decarboxylase gene (*cadA*). Probable alteration of the *Plesiomonas cadA* gene could be responsible for the virulence of some strains. Deletion of the *cadA* gene in *Shigella* reportedly promotes virulence to evade host cell protection against *Shigella* enterotoxin that relies on the inhibition of cadaverine synthesis (Maurelli et al., 1998; Brosch et al., 2001). Virulence plasticity is attributed principally to insertions and deletions in a pathogen genome (Brosch et al., 2001). *Plesiomonas* harbors IS elements that share homology with those found in pathogenic bacteria. The identified *Plesiomonas* IS elements could have selected for the pathogenic potential of this microorganism in many instances. IS elements are known mutagenic systems of the bacterial genome that modify genes by deletion, disruption, or up-regulation of neighboring genes (Mahillon and Chandler, 1998; Gyles and Boerlin, 2014). The identified IS elements might have impacted the evolutionary advantage of virulence in *Plesiomonas*, with both diarrheagenic and extraintestinal pathogenesis of this microorganism increasingly emerging.

The prophages detected in *Plesiomonas* strain genomes contribute to their genetic identity and possible differences of virulence traits. Prophage entities are known to impact bacterial pathogen fitness, from increasing pathogenicity potential, to chromosomal rearrangement and prophage-encoded VFs (Canchaya et al., 2004; Bondy-Denomy and Davidson, 2014).

The observed numerical and structural variation in *Plesiomonas* CRISPR elements might be invaluable for strain typing. CRISPR variability is employed as a gold standard for strain-typing purposes in epidemiological studies of *Mycobacterium tuberculosis* (Kamerbeek et al., 1997; Comas et al., 2009; Abadia et al., 2010; Gomgnimbou et al., 2012), *Campylobacter jejuni* (van Belkum et al., 2001; Schouls et al., 2003; Louwen et al., 2013*), Erwinia amylovora* (Rezzonico et al., 2011), *Salmonella* (Liu et al., 2011), *Propionibacterium acnes* (Bruggemann et al., 2012), and *Corynebacterium diphtheria* (Mokrousov et al., 2007, 2009). Further, the discriminatory power of CRISPR typing techniques is enhanced by combining with multilocus sequence typing and amplified-fragment length polymorphism analyses (Schouls et al., 2003), CRISPR-MVLST (Shariat et al., 2013), or spoligotyping (Mokrousov et al., 2009; Ginevra et al., 2012). In addition, CRISPR-Cas systems have been adopted for bacterial virulence profiling and resistance studies of enterococci (Frimodt-Moller, 1993; Bourgogne et al., 2008; Palmer and Gilmore, 2010; van Schaik et al., 2010; Yosef et al., 2012; Tremblay et al., 2013), *Salmonella* (Sheikh et al., 2011), *S. aureus* (Kinnevey et al., 2013; Otto, 2013), enterohemorrhagic *E. coli* (Delannoy et al., 2012), *C. jejuni* (Louwen et al., 2013), *Streptococcus pyogenes* (McShan et al., 2008; Nozawa et al., 2011), and *Mycoplasma gallisepticum* (Delaney et al., 2012). Generally, studies revealed that type II CRISPR-Cas–harboring species, including the genera *Campylobacter, Neisseria*, and *Streptococcus*, exert tremendous pressure on the food industry and healthcare system (Louwen et al., 2014). Therefore, in-depth exploration of CRISPR systems might enable strain delineation and pathotyping in *Plesiomonas*.

The absence of plasmids in the four strains revealed that most virulence traits were chromosomally borne. Nevertheless, presence of plasmids and plasmid-borne traits have been reported in some *P. shigelloides* isolates (Herrington et al., 1987; Fischer et al., 1988; Olsvik et al., 1990; Kelly and Kain, 1991; Avison et al., 2001; Al-Gelawi and Al-Jeboory, 2011; Abdelhamed et al., 2018).

## Conclusion

As demonstrated in the current study, comparative genomics of *Plesiomonas* strains revealed the presence of probable virulence traits that could not be unambiguously determined using traditional approaches. The remarkable homology or orthology of *Plesiomonas* ORFs with VFs and antibiotic resistance genes from species commonly identified in the healthcare and food industries are non-accidental and further attest to the pathogenic capacity of plesiomonads. We hypothesize that differential expression and regulation of virulence traits in the presence of different host factors or under different conditions might be responsible for the lack of reproducibility (i.e., dissimilar results) of data on *Plesiomonas* pathogenicity obtained during early investigations that relied on conventional experimental models. That probably stems from the facts that the conditions that warrant the (over)expression of a *Plesiomonas* virulence gene in the host might not be replicable under experimental conditions, compromising the efficiency of conventional protocols for the assessment of the bacterial virulence potential. Expression of such virulence genes or validation of our hypothesis might be of interest to future transcriptomic investigations. Further, the current study provides clues on an alternative exploratory approach that could be adapted for strain typing and delineation of pathogenic and non-pathogenic variants of the bacterium. In-depth exploration of numerical and structural variation of CRISPR systems in *Plesiomonas* in combination with other specific high-throughput techniques could provide low-cost but sufficient discriminatory power invaluable for strain typing and virulence profiling. Thus, the quest for developing rapid and inexpensive tools for strain and diagnostic characterization of *Plesiomonas* becomes imperative. We conclude that *P. shigelloides* possesses some VFs associated with gastroenteritis and extraintestinal infections; however, the role of host factors in the onset of infections cannot be undermined.

## Data Availability Statement

All datasets generated for this study are included in the manuscript and the Supplementary Files.

## Author Contributions

TE conceptualized idea for the study, responsible for data collection, analysis, interpretation, and wrote the first draft of the manuscript. AO participated in result interpretation, proofread the first draft, and supervised the study. All authors read and approved the final manuscript.

## Conflict of Interest Statement

The authors declare that the research was conducted in the absence of any commercial or financial relationships that could be construed as a potential conflict of interest.

## Footnotes

1 http://bioinformatics.psb.ugent.be/webtools/Venn/

2 https://www.ncbi.nlm.nih.gov/genome/genomes/17449?

## Supplementary Material

FIGURE S1 A Venn diagram showing distribution of IUSRVTs in four *Plesiomonas shigelloides* strains. **302-73, GN7, LS1, and NCTC10360** **(9)**: multispecies ubiquinol-cytochrome c reductase iron-sulfur subunit, multispecies iron donor protein *CyaY*, multispecies iron-sulfur cluster insertion protein ErpA, multispecies succinate dehydrogenase/fumarate reductase iron-sulfur subunit, zinc/iron-chelating domain-containing protein, iron export ABC transporter permease subunit *FetB*, iron-sulfur cluster repair di-iron protein, ferrous iron transport protein B, multispecies iron-sulfur cluster assembly protein IscA; **GN7, LS1, NCTC10360** **(1):** iron ABC transporter permease; **302-73, LS1 and NCTC10360** **(2):** multispecies zinc/iron-chelating domain-containing protein and iron transporter FeoA; **302-73, GN7 and LS1 (2):** multispecies succinate dehydrogenase iron-sulfur subunit and multispecies iron-sulfur cluster scaffold-like protein; **302-73 and LS1 (1)**: iron(III) ABC transporter ATP-binding protein; **NCTC10360 (2)**: succinate dehydrogenase iron-sulfur subunit; GN7 (1): multispecies iron transporter *FeoA*; **302-73 (1):** iron(III) ABC transporter permease.

FIGURE S2 A Venn diagram showing distribution of HUSRVTs in four *Plesiomonas shigelloides* strains. **302-73, GN7, LS1 and NCTC10360 (4):** heme ABC transporter ATP-binding protein, heme exporter protein CcmB, heme utilization protein HutZ, heme ABC exporter ATP-binding protein CcmA; **GN7,** **LS1 and NCTC10360 (1):** putative heme utilization radical SAM enzyme HutW; **302-73, GN7 and LS1 (2):** biliverdin-producing heme oxygenase and multispecies: heme exporter protein CcmD; **302-73 and NCTC10360 (1):** heme exporter protein CcmC; **GN7 and LS1 (2):** heme ABC transporter permease and heme peroxidase; **NCTC10360 (2):** heme exporter protein CcmD.

DATA SHEETS S1–S4 Detailed VRprofile query outputs for the four *P. shigelloides* genomes.

DATA SHEET S5 Structural and compositional details of CRISPRs obtained in *P. shigelloides* strain.

## Abbreviations

AR: acquired antibiotic resistance determinant
ARI: antibiotic resistance island
CRISPR: clustered regularly interspaced palindromic repeats
GI: genomic island
HUSRVT: heme uptake system related virulence trait
ICE: integrative and conjugation element
IS: insertion sequence
IUSRVT: iron uptake system related virulence trait
ORF: open reading frame
PAI: pathogenicity island
PVF: putative virulence factor
TSE: type secretion effector
TSS: type secretion system
VF: virulence factor
