# Supplementary figures and images for: Pathogenomics of Virulence Traits of Plesiomonas shigelloides That Were Deemed Inconclusive by Traditional Experimental Approaches
Source: Front Microbiol. 2018 Dec 21;9:3077. doi: 10.3389/fmicb.2018.03077 (PMC6309461; doi:10.3389/fmicb.2018.03077)

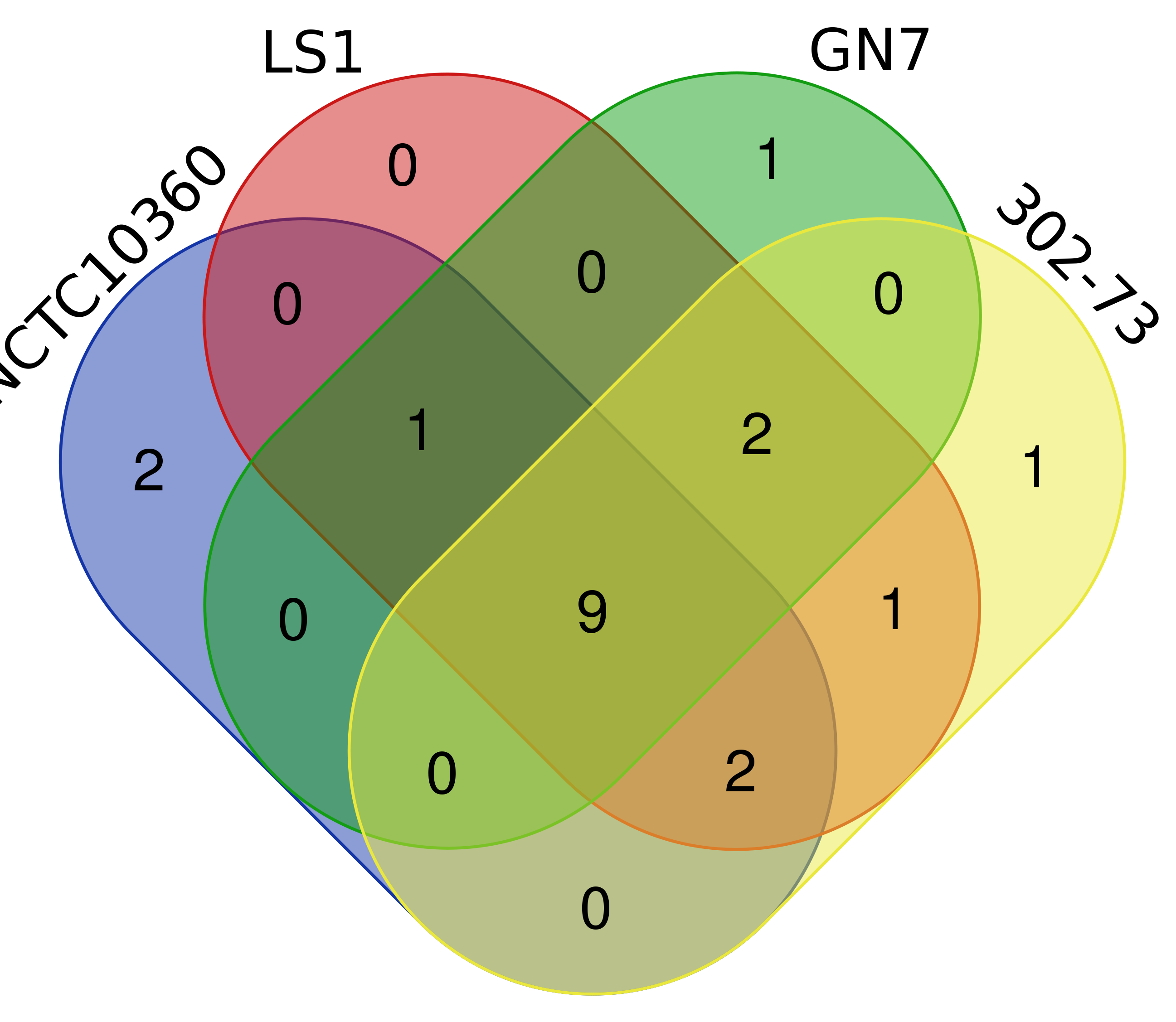

Supplement: FIGURE S1 — A Venn diagram showing distribution of IUSRVTs in four Plesiomonas shigelloides strains. 302-73, GN7, LS1, and NCTC10360 (9): multispecies ubiquinol-cytochrome c reductase iron-sulfur subunit, multispecies iron donor protein CyaY, multispecies iron-sulfur cluster insertion protein ErpA, multispecies succinate dehydrogenase/fumarate reductase iron-sulfur subunit, zinc/iron-chelating domain-containing protein, iron export ABC transporter permease subunit FetB, iron-sulfur cluster repair di-iron protein, ferrous iron transport protein B, multispecies iron-sulfur cluster assembly protein IscA; GN7, LS1, NCTC10360 (1): iron ABC transporter permease; 302-73, LS1 and NCTC10360 (2): multispecies zinc/iron-chelating domain-containing protein and iron transporter FeoA; 302-73, GN7 and LS1 (2): multispecies succinate dehydrogenase iron-sulfur subunit and multispecies iron-sulfur cluster scaffold-like protein; 302-73 and LS1 (1): iron(III) ABC transporter ATP-binding protein; NCTC10360 (2): succinate dehydrogenase iron-sulfur subunit; GN7 (1): multispecies iron transporter FeoA; 302-73 (1): iron(III) ABC transporter permease. [file Image_1.TIF]

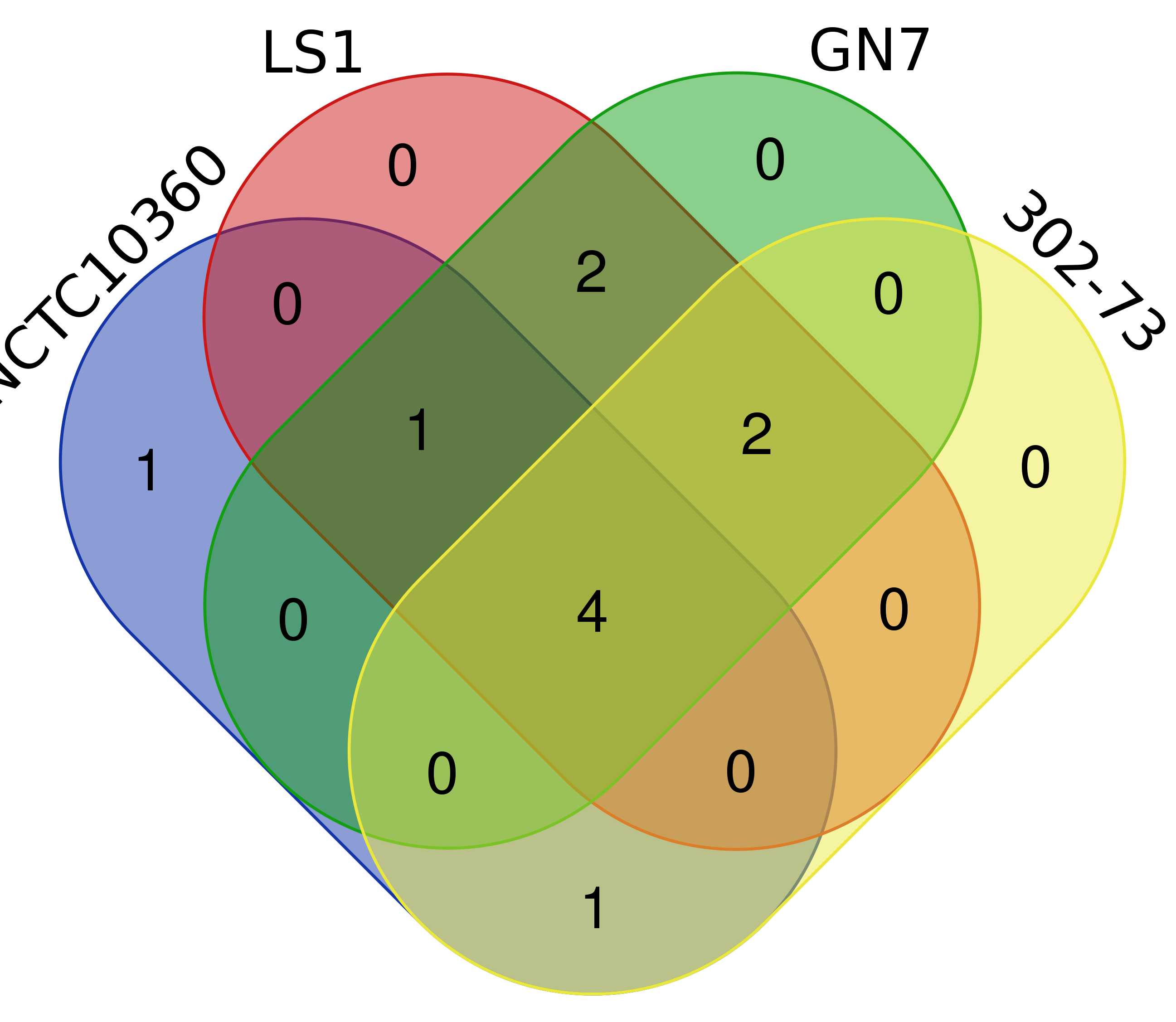

Supplement: FIGURE S2 — A Venn diagram showing distribution of HUSRVTs in four Plesiomonas shigelloides strains. 302-73, GN7, LS1 and NCTC10360 (4): heme ABC transporter ATP-binding protein, heme exporter protein CcmB, heme utilization protein HutZ, heme ABC exporter ATP-binding protein CcmA; GN7, LS1 and NCTC10360 (1): putative heme utilization radical SAM enzyme HutW; 302-73, GN7 and LS1 (2): biliverdin-producing heme oxygenase and multispecies: heme exporter protein CcmD; 302-73 and NCTC10360 (1): heme exporter protein CcmC; GN7 and LS1 (2): heme ABC transporter permease and heme peroxidase; NCTC10360 (2): heme exporter protein CcmD. [file Image_2.TIF]
