# Supplementary material for: Pathogenomics of Virulence Traits of Plesiomonas shigelloides That Were Deemed Inconclusive by Traditional Experimental Approaches
Source: Front Microbiol. 2018 Dec 21;9:3077. doi: 10.3389/fmicb.2018.03077 (PMC6309461; doi:10.3389/fmicb.2018.03077)
Supplement: DATA SHEET S5 — Structural and compositional details of CRISPRs obtained in P. shigelloides strain. [file Data_Sheet_5.docx]

Table S5. Structural or compositional details of CRISPRs obtained in *P. shigelloides* strains.

| Start | Repeat | Spacer | Spacer Length |
| --- | --- | --- | --- |
|  | NCTC10360 CRISPR_1 (634986 - 635157)/ 172/ | |  |
| 634986 | TCAGTACGCTGCTGCTGGCGTTGCA | ACAGTCGCTCTGTGGCTTGCTGTAGGCGATGTTGTAAATCATCCAGCTGCATC | 53 |
| 635064 | TGCTGGCGCTGTAGACGCGCTTGTG | GGTGTTGGCGAAGTAAACGGTGCTGTAAGCGCTCCAGATGCTGC | 44 |
| 635133 | TGCTGGCGCTGTAGCTGGCGTATCG |  |  |
|  | NCTC10360 CRISPR_2 | |  |
| 681979 | CAACACCACCAAAAACGGGC | TTGTTTTAATACCACCAAAAACGGTGCTGTTAATAAAGA | 39 |
| 682038 | GAACACACCCAAAAACGGTA | ACGTTACCGAAAACGGGTGTGTTAACCAAAT | 31 |
| 682089 | GAACACTACCGAAAACGGTA | CTGTTACTGAGGTAAATACACCCAATTTTGGAGCC | 35 |
| 682144 | AAAGACACCCAAAAACGGGG |  |  |
|  | Plesiomonas shigelloides 302-73 | |  |
| 488056 | CTATTTTTATTTCATCCACACCCA | GTTTTTGGATATAGGAAAACCCACCATAAC | 30 |
| 488110 | CTGTGCCAAAATCATCCAATTTAA | ATCGATAACCAAACAGCTTTATTTTAGCTATTTCTCGCGCTG | 42 |
| 488176 | CAGTTTCAAAATCATCGACTTTAA |  |  |
|  | 2 | |  |
| 698058 | CAGCGTTCAGCGCCGCTTTCA | GGCAACGCAGAGCCATCGGGCTGTTACGCAGCATTTCACGGCAC | 44 |
| 698123 | CAGCGCACGGTTTCGCGCTCC | AGCTGCTCCAGTGGAACAACAGTGTTCACCAGACCCATGTC | 41 |
| 698185 | CAGCGCTTGCTGCGCATCGTA |  |  |
|  | 3 | |  |
| 1317065 | TCGCGACGCTCACGACGCTCGCCGCTGT | GGCTACGCTCACCGCTACGACGCTCACCGTTGCCAAAGCTACCGCGTGGACGG | 53 |
| 1317146 | TCACCGCCTTCACGACGTGGACGATCAC | TGCCTTCACGGCGTGGACGGTCACCACCTTCACGACGCGGACGA | 44 |
| 1317218 | TCACCACCTTCGCGACGTGGACGATCAC |  |  |
|  | 4 | |  |
| 1317501 | CGTCCATTGGGCCCACATCACGACG | CTCACGACGCTCAGGACGATCGCCAT | 26 |
| 1317552 | CACGACGTGGACGGTCATCACGACG | ACCATCACGGCTGTCACGACGGTCACGACCACCGCGCT | 38 |
| 1317615 | CATCACGGTCACGGAATTCACGACG |  |  |
|  | 5 | |  |
| 2287008 | CTATTTTTATTTCATCCACACCCA | GTTTTTGGATATAGGAAAACCCACCATAAC | 30 |
| 2287062 | CTGTGCCAAAATCATCCAATTTAA | ATCGATAACCAAACAGCTTTATTTTAGCTATTTCTCGCGCTG | 42 |
| 2287128 | CAGTTTCAAAATCATCGACTTTAA |  |  |
|  | 6 | |  |
| 2497010 | CAGCGTTCAGCGCCGCTTTCA | GGCAACGCAGAGCCATCGGGCTGTTACGCAGCATTTCACGGCAC | 44 |
| 2497075 | CAGCGCACGGTTTCGCGCTCC | AGCTGCTCCAGTGGAACAACAGTGTTCACCAGACCCATGTC | 41 |
| 2497137 | CAGCGCTTGCTGCGCATCGTA |  |  |
|  | 7 | |  |
| 3116017 | TCGCGACGCTCACGACGCTCGCCGCTGT | GGCTACGCTCACCGCTACGACGCTCACCGTTGCCAAAGCTACCGCGTGGACGG | 53 |
| 3116098 | TCACCGCCTTCACGACGTGGACGATCAC | TGCCTTCACGGCGTGGACGGTCACCACCTTCACGACGCGGACGA | 44 |
| 3116170 | TCACCACCTTCGCGACGTGGACGATCAC |  |  |
|  | 8 | |  |
| 3116453 | CGTCCATTGGGCCCACATCACGACG | CTCACGACGCTCAGGACGATCGCCAT | 26 |
| 3116504 | CACGACGTGGACGGTCATCACGACG | ACCATCACGGCTGTCACGACGGTCACGACCACCGCGCT | 38 |
| 3116567 | CATCACGGTCACGGAATTCACGACG |  |  |
|  | strain LS1 | |  |
| 297760 | TGCGCATGGAAAAATGCGTAGA | AGCATTCAATAACCATATTTCCAAAATCCGTACCGGCCG | 39 |
| 297821 | TGCGCATCCTAGCCTGCTGGAA | GGGATCACCGTTGAATATTACGGCTCACCAACTCCGC | 37 |
| 297880 | TGCGCCAGCTGGCCAACGTGGT |  |  |
